# Experiences of supporting primary and community healthcare workers affected by domestic abuse in the United Kingdom: A cross-sectional survey

**DOI:** 10.1080/13814788.2025.2571600

**Published:** 2025-11-10

**Authors:** Sandi Dheensa, Gene Feder, Christian Mallen, Alison Gregory

**Affiliations:** ^a^ BRIGHT Group, Centre for Academic Primary Care, Bristol Medical School, Population Health Sciences, University of Bristol, Bristol, UK; ^b^ School of Medicine, Keele University, Keele, Staffordshire, UK

**Keywords:** Primary health care, general practice, domestic violence, intimate partner violence, community health services

## Abstract

**Background:**

Healthcare workers are expected to identify and respond to domestic abuse among patients. However, research has neglected healthcare workers’ own experiences of domestic abuse.

**Objectives:**

Focusing on UK primary and community healthcare workers with formal support roles (e.g. line managers, human resources, employee assistance professionals), this exploratory study aimed to illustrate workplace support offered to healthcare colleagues affected by domestic abuse.

**Method:**

We used an online cross-sectional survey with closed and qualitative free-text questions, advertised via mailing lists and social media, targeting healthcare workers. Our mixed methods embedded design involved quantitative descriptive analysis with content analysis of qualitative free text to explain and interrogate results.

**Results:**

Sixty-two people in healthcare roles supporting colleagues responded, mostly from community hospitals, dentistry, and general practice. Few workplaces had staff domestic abuse policies. Support measures were limited. Emotional support, signposting, and adjusted working hours were the most common types of support available. Training on supporting affected colleagues was rare. Few environments had specialist domestic abuse advocates who supported staff. Along with needing policies, training, and in-house support, respondents indicated a need for a cultural shift to address myths about domestic abuse and hierarchical power, particularly regarding healthcare workers who perpetrate abuse.

**Conclusion:**

Despite several limitations, including substantial missing data, our work highlights that primary and community healthcare workplaces should explore the implementation of practical and emotional support measures; healthcare-based domestic abuse advocates with staff support; and training on supporting colleagues. Further pan-European comparative research should surface good practice and foster cross-learning.

## Introduction

Over 20% of England and Wales’ population has experienced domestic abuse (DA) [1]. DA has long-lasting, wide-ranging health consequences [2,3] and cost the economy £66bn in one year, including over £2 m in healthcare service costs and £14 m in lost output [4]. Policies [5,6] urge healthcare workers (HCWs) to enquire about DA and refer survivors (of DA), with consent, for specialist support. Specialist DA advocates are available in some hospitals [7] and via a programme called ‘IRISi’ in general practice (GP) and sexual health, which has also been trialled elsewhere (e.g. dentistry) [8].

HCWs’ own experiences of DA have been under-researched. A recent meta-analysis of studies including from UK, Italy, Spain, Finland, and Sweden, showed a high proportion have lifetime experience: 31.3% overall (41.8% of women, 14.8% of men) [9]. DA among HCWs is therefore a concern across international healthcare systems. A 2016 England and Wales study showed 14% of female nurses, midwives and healthcare assistants experienced past-year DA, which was triple the general population prevalence [10]. A 2021 UK study [11] found that female doctors faced unique barriers to disclosure and support-seeking, e.g. stigma (‘I should have known better’) and fears their regulatory body would be told. A few disclosed to a particular person or team, which helped them to cope: disclosure was safer for patient care because it alerted colleagues to the increased potential for clinical errors. Neither the meta-analysis nor subsequent international research has highlighted specific DA interventions for HCWs.

Workplaces can increase the likelihood of survivors ending relationships with perpetrators, e.g. via (in)formal support, financial benefits, increased agency, and respite from the perpetrator. Colleagues can provide helpful responses to DA disclosures, e.g. support, resources, and referrals [12]. Paid DA leave, not a statutory right in England, Wales, or Scotland, is available to at least some employees in Northern Ireland, Republic of Ireland, Italy, Slovenia, Australia, Canada, and New Zealand [13]. However, workplaces can also compound abuse and trauma e.g. via judgement, workplace conflict, and workplace violence, which affect healthcare staff at high rates [12]. Perpetrators might be survivors’ colleagues [11]. Line managers (individuals who directly manage employees), occupational health (OH) teams, human resources (HR), and employee assistance programmes (EAPs) have important roles in supporting survivors, e.g. connecting them to advocacy and/or psychotherapy, which can improve mental health and reduce re-victimisation [14,15].

There have been multiple calls for research to enable employers to better understand workplaces’ roles in increasing survivors’ safety and well-being [12,16]. Hospitals in Victoria, Australia, have embedded government-funded ‘family violence workplace support programs^1^, with dedicated contact officers (existing hospital staff who provide information about workplace support), specific (although non-mandatory) manager training about the impact of DA on work, and partnerships with external support organisations for employees. The UK National Health Service (NHS) has lagged behind, although positive policy changes have emerged [6,17]. In 2017, NHS Employers (which supports/represents NHS workforce leaders/employers) [18] published guidance for hospitals wishing to develop local staff DA policies, but 2 years later [19] only 68% of secondary care trusts and health boards (which oversee groups of geographically close hospitals) had a local policy. Only two policies included all the guidance document’s recommendations. No equivalent research has surveyed primary and community healthcare. More recently, sexual misconduct policies have been published following reports of high prevalence [20,21]: sexual violence often happens within a DA context [1].

Despite these policy advances, no research explores workplace DA support for UK HCWs. We aimed to fill this gap. We built on earlier [11] doctor-specific work by exploring a larger group of HCWs’ experiences, focusing mainly on primary healthcare (including GP, dentistry, and pharmacy) and community healthcare (including extensive, diverse services delivered in patients’ homes, care homes, community clinics, community centres, schools, and hospices). These HCWs have multiple opportunities to engage with perpetrators and survivors as patients [22,23].

### Aim

Focusing on UK HCWs with formal support roles (e.g. line managers, HR, and EAP staff), this exploratory study aimed to illustrate support offered to healthcare colleagues affected by DA.

## Methods

### Design

This article presents PRESSURE study findings (www.bristol.ac.uk/pressure-study) comprising a cross-sectional survey with closed and free-text questions. We used a mixed-methods embedded design [24]. The wider study explored HCWs’ own experiences of DA, as well as experiences of those who might support them, and local staff DA policies.

### Survey development

The study team (academics, clinical academics, and lived experience advisors [four HCW-survivors]) co-developed the survey. The process involved item pooling and refining by SD and AG who then sent the team a draft for feedback. Some gave written feedback; we also held a meeting to discuss content and wording. Feedback included providing free-text boxes for complex questions.

The survey’s participant information pages provided a DA definition: respondents could self-define as survivors. We report survivor data elsewhere [25]. The first 26 questions (part 1) were about respondents’ own experiences of DA; the subsequent 15 questions (part 2) were about experiences of supporting others. Respondents could answer as a survivor (completing part 1), a supporter of others (completing part 2), or both (completing parts 1 and 2). A few questions featured in both parts: branching logic and instructions directed respondents to skip questions they had already answered in part 1. The survey was transferred to REDCap, an online platform. Fifteen people, including the four HCW-survivors from our study team, piloted the online survey including participant information, giving final feedback e.g. on the clarity of safe completion instructions, layout, and completion time. Supplementary Appendix 1 contains safeguarding-relevant extracts from the participant information that respondents with personal experience saw. (Those completing the survey as supporters saw similar information adapted for context.) These pages, and the final page, provided details for a range of DA support services, including for those worried about their own behaviour. The message that respondents could have a break and use the ‘save and return later’ function was repeated on each page of the survey. The final survey (see Supplementary Appendix 2) was open from 09/2022 until 12/2022, launched after receiving approval from University of Bristol Research Ethics Committee [reference 1139]. Written consent was documented within the survey.

### Recruitment

The survey and/or study website was advertised via directors of primary healthcare networks (groups of general practices working closely together), the study’s Twitter profile (tagging accounts with large followings, e.g. Royal Colleges), a dentistry staff DA training session, a regional pharmacist networking event, and a GP journal article [26]. Wide advertising precluded response rate estimation.

An exclusion criterion was working outside the UK, which our survey advertising made explicit. Our primary and community healthcare focus was also explicit. However, we chose not to exclude HCWs from other areas, e.g. integrated care boards (ICBs), which are responsible for planning and funding local NHS services, as their responses enhanced the findings.

### Missing data

Substantial data is missing throughout the survey: we report missing data for each section (Supplementary Appendix 3 has detail). We removed this data from frequency calculations. The pattern of missing data suggests attrition: i.e. people dropped out of the survey partway through. Most who dropped out had already completed part 1 as survivors. The ‘save and finish later’ option appeared under-utilised despite our instructions for its use. We chose not to mandate answers to all questions to give respondents (particularly survivors) a choice about answering questions. We chose not to handle missing data using an analytic approach, such as imputation, given that our aim was to be descriptive and exploratory.

### Outcomes

Survey items asked about staff DA policies, workplace support measures (adapted from NHS England policy, NHS Employers’ guidance [18], and other individual policies analysed in the wider study), training on supporting colleagues, specialist DA workers, resources, and experiences supporting colleagues.

### Analysis

Analysis comprised quantitative descriptive analysis. Basic content analysis [27] of qualitative free-text (which is descriptive, systematic, centres quantitative results, and relies partially on frequency counts of low-inference text that does not necessitate extensive interpretive judgments) helped to explain results. We used survey items to create an *a priori* code list (remaining open to inductively generated codes), repeatedly reading text to reduce it to these codes. SD led the analysis, analysing quantitative data within REDCap, but discussed qualitative findings with co-researchers to revise the code list and agree on a final analysis. We illustrate qualitative findings with anonymised verbatim quotations and allocate ID codes corresponding to survey respondent IDs. Co-authors checked analysis.

## Results

### Respondent roles

Sixty-two people with roles involving support of staff affected by DA (‘staff-survivors’) partially completed or completed the survey – 40 were themselves staff-survivors. Areas of work were community hospital (*n* = 24), dentistry (*n* = 14), GP (*n* = 12), ICB (*n* = 4), ambulance service (*n* = 2), and DA advocacy (straddling DA service and sexual health/GP) (*n* = 2). There was one community social worker, public health (screening and immunisations) HCW, trade union representative, and acute hospital HCW: the latter drew on previous workplace experiences. Supplementary Appendix 4 breaks down key results by the most common sectors.

Most had line management or safeguarding duties (i.e. had expertise or provided leadership around safeguarding children or adults at risk, e.g. disabled adults); *n* = 5 worked in HR (two as ‘business managers’ in GP, two for dental practices, and one in a community hospital), *n* = 2 worked in EAPs, *n* = 2 were healthcare-based DA workers (as stated above), and one worked in community hospital OH.

### Presence of staff DA policies

Most respondents, 20/44, did not know whether their workplace had a staff DA policy; 14/44 reported it did not; and only 10/44 reported that it did (18 records missing). Table 1 shows the spread across areas: community hospital or services made up the greatest proportion of areas with a policy.

**Table 1.** Staff domestic abuse policies in place (*n* = 44).

Area	Yes	No	I don’t know
Community hospital or service	7		9
General Practice		5	5
Dentistry		6	2
Integrated Care Board (commissioner)	1	1	2
Public health (screening and immunisations)			1
Union	1		
Sexual health		1	
Ambulance service	1		
Acute trust		1	
Domestic abuse advocate			1
**Total**	**10**	**14**	**20**

Free-text comments from respondents without staff DA policies included one from a GP line manager[R1], who explained, *“there are no formal policies other than standard employment practices; each case is managed on its own basis”*. Completing the survey encouraged respondent reflection, with some respondents identifying that the offered support was *“not enough”* (EAP counsellor[R340]). A GP business manager[R32] wrote, “*We don’t have a policy, but I’ll put one into place now: we’re a very caring employer and all staff are aware of the open-door policy: staff can come and talk about anything in confidence”.*

Of those who had a staff DA policy, *n* = 2 reported it was part of another policy (e.g. patient DA policy). Moreover *n* = 7 felt that employee awareness of the policy was possibly low. A few explained their answers:

*“My OH team is definitely aware—I’m not sure whether all line managers would be aware.”* (Community hospital line manager[R39])

“It’s been advertised, but staff turnover negates this. It is available on the intranet.” (ICB designated safeguarding nurse[R56])

“I endeavoured to get senior management to publicise the policy and was informed they wouldn’t do it because it was about a negative issue! I haven’t tried again—I probably should.” (Union representative[R26])

A few respondents were developing new policies at the time of their survey response. One private (i.e. non-NHS) dentistry HR respondent[R213] reported that as practice owners, they had leeway on devising policies: they wanted to implement a new policy “*that outlines the process of dealing with someone who is undergoing abuse rather than being reactive…[with] a written return-to-work process in case they have more than 12 months off”.* Their new policy had been prompted by an employee who experienced DA and underwent a generic (and possibly inappropriate) return-to-work process following sick leave. Additionally, a GP line manager[R5] reported that they were drafting a policy taking account of different staff needs, including non-current DA.

A few articulated the insufficiency of policies alone: one ICB EAP counsellor[R340] wrote, *“I find the NHS has policies so they can “tick the box” but in practice, they often aren’t applied”.*

### Support measures in place

When asked whether recommended support measures (e.g. from NHS Employers’ guidance) were in place, whether in a written policy or not, affirmative responses were low for all support measures. Table 2 summarises available measures and numbers of respondents who reported them as available. As illustrated, work pattern changes, special leave, and permission to attend appointments were most common. Eighteen respondents additionally stated that they offered the right to confidentiality. Waiving sickness absence management was uncommon despite NHS Employers’ guidance recommending it. Paid leave was also uncommon, likely because it is not statutory outside Northern Ireland.

**Table 2.** Support measures in place (*n* = 62).

Support measure	Number of respondents who reported this support measure was available
**Working patterns**	
Changes to working times, days, or patterns	12
Changes to specific duties (e.g. to avoid contact with perpetrators)	9
Option for redeployment or relocation	6
Waiving the use of informal and formal sickness absence management stages when sickness might be domestic abuse-linked	4
**Leave**	
Special leave provisions (e.g. using existing leave, or the option for unpaid leave)	12
Permission to attend appointments related to domestic abuse during work hours	12
Permission to use private spaces at work to hold domestic abuse-related appointments	7
Paid leave for domestic abuse	4
**Safety planning**	
Workplace safety measures e.g. blocking emails, screening phone calls, reception and security alerted that perpetrator might come to the workplace	9
Permission to use work phones or computers to look up information and access support	9
Review of personal information held by workplace e.g. address	6
Measures to ensure safety while travelling to and from work	5
Option to stay at work for safety (e.g. to stay late or to sleep at work)	5
Training for security and reception staff on managing perpetrator who turns up at workplace	5
**Referrals**	
Occupational health referral	11
Support from qualified professionals (e.g. staff counsellors or therapists)	10
Referral to an employee assistance program	6
Signposting to an in-house domestic abuse advocate	5
**Pay**	
Changes to pay arrangements	7
Referral to a credit union or financial advisory service	4

### Reflections on support offered

Free-text comments about the support respondents or their workplaces offered ranged from thinking the support was *“good”* or *“excellent”* (these respondents worked in organisations with most of the listed options). Additionally, an ambulance service safeguarding lead stated, *“we provide good support and are flexible to needs of individuals*”[R392] (despite indicating that only EAP and OH referrals were available). Similar to this respondent, a GP line manager indicated an individualised approach: *“We are a very small organisation; we’ve employed a much less policy [focused] and standardised approach to [affected] staff. We don’t have written policies and actually don’t feel a need for this: we feel we have a culture for how we relate to staff that responds to all the things that relate to being a human and a part of our team.”* Regarding sickness absence, they commented *“we don’t run formulaic sickness rules: it’s far more person-centric so accommodates specific issues to the individual”*[R11]. The small size of the organisation enabled a personalised approach.

However, not everyone felt their organisation offered adequate support: *“Due to a restructure, the HR function has been centralised and I would be concerned about its accessibility and timeliness*” (Community nurse, line manager[R50]).

### Training

Regarding training on supporting staff-survivors, 34/45 indicated no training and 9/45 indicated that it was included in broader training (17 records missing). This training was mostly one-off (*n* = 4), with some repeated every 3 years (*n* = 3) or annually (*n* = 2). An additional *n* = 2 reported specific training, which they sought themselves and could repeat when needed: one community hospital respondent[R39] wrote, *“In my role in OH, I have a responsibility to ensure I know what DA looks like and how to support staff appropriately, including safeguarding”.* Similarly, a GP line manager[R11] wrote, *“It’s an area of interest so I did it for my own training needs”.*

Training was self-directed e-learning (*n* = 5), online live training (*n* = 4), in-person live training (*n* = 1), or unspecified (*n* = 1). Training length varied: self-directed was 0.5 to 3 h; live training was 2 to 8 h.

### Specialist workers

On the provision of specialist DA workers with designated roles to support patients, 11/47 respondents indicated that they did not know whether their workplace had such a role, 18/47 indicated no provision, and 18/47 reported provision (15 records missing). Of the 18 that reported provision, 6/18 indicated that the worker supports staff-survivors, 6/18 reported that they do but not as an official part of their role, 3/18 reported that they do not support staff, and 3/18 were unsure.

### Resource materials such as posters

Regarding whether resource materials about DA (e.g. posters or leaflets) were available in the workplace, 20/44 reported they were, 10/44 reported they were not, 10/44 were unsure, and 4/44 said ‘other’. (18 records missing). Of the 20 who reported that resources were available, 12/20 indicated they were well-displayed and accessed by staff, 6/20 felt they were poorly displayed, and 2/20 reported well-displayed resources not accessed by staff.

The four who chose ‘other’ indicated in free text that their resources were for staff *and* patients (dental hygienist), resources were displayed but whether staff accessed them was unclear (GP line manager), and that resources had safeguarding – but not specific DA – information (GP line manager). A dental practice manager (also a survivor) was developing staff DA resources herself.

### Experiences of providing support

On whether they had encountered staff-survivors in the past 5 years, 16/34 respondents had and 18/34 had not (28 records missing). Most had encountered just 1 or 2 staff-survivors; one ambulance service respondent had encountered 30 to 50. The 16 respondents detailed the actions they took for staff-survivors in free text, which Table 3 categorises and presents with illustrative quotations. Whereas Table 2 shows awareness of what support was ‘officially’ available, Table 3 shows what actions respondents took (which in some cases was shaped or limited by mechanisms ‘officially’ available). All actions were positive; however small numbers chose each action, suggesting that inaction was common.

**Table 3.** Action taken for affected staff with free-text examples (*n* = 16).

Emotional support and listening	5
Signposting to domestic abuse organisation	4
Safety planning for home and work *Offered practical support such as lifts to school for children and accompanied her to get new mobile phone. Supported with time out of work for appointments with GP [General Practice], social care and legal team.* (Community hospital safeguarding lead [Respondent 47]). Other examples: enabling use of workspace to make calls, supporting staff to not have phone in work if being harassed, enabling document storage at work.	4
Adjusted working hours	3
Time off and different types of leave including ‘unauthorised leave’	3
Regular one-to-ones or check-ins with someone with whom they have rapport	2
Sickness absence managed differently	2
Risk assessment *We risk assess each case and put measures in place to protect staff. We’ve reported some to the Medical Protection Society or supported staff to do so, as well as directing to support services. We purchased 50 [safety] apps for staff who feel it’d be useful for tracking and recording ongoing domestic abuse.* (Ambulance service safeguarding lead [Respondent 392])	2
Consideration of safeguarding needs	2
Referral to free counselling	2
Referral to domestic abuse organisation	1
Referral to occupational health with permission	1
Security measures: preventing perpetrators attending workplace	1
Help to access financial support via grants e.g. via charities	1
Crisis support *A staff member had a crisis [in public] and phoned in: she didn’t know where she was. We talked to [someone nearby by phone], arranged to pick her up, and accompanied her to her own GP [General practitioner]* (General Practice line manager [Respondent 11]).	1

Of support provided, emotional support and listening was most common, followed by signposting and safety planning. Help to access financial support was least commonly provided (as well as least commonly available as Table 2 shows). Moreover, referrals to different services were uncommon, suggesting a preference to signpost staff-survivors rather than directly refer them. Specific actions reported in free text included risk assessing each situation, purchasing and providing personal safety apps for all staff, practical support (e.g. organising school transport for children and providing a new mobile phone), offering space to work on-site during the COVID-19 pandemic, and providing crisis support.

### Support to support others

The 16 respondents who had encountered staff-survivors mostly (*n* = 12) received support for themselves, e.g. around what to say or do for staff-survivors. They received support from an external DA service (*n* = 5), workplace safeguarding team (*n* = 5), the police (*n* = 4), HR (*n* = 4), EAP (*n* = 3), an organisational development business partner (*n* = 1), healthcare-based DA worker (*n* = 1), external HR (*n* = 1), or elsewhere (*n* = 3). In general, respondents reported *“good”* support. One community hospital line manager[R39] who sought support from her workplace safeguarding leads, workplace DA advocate, *and* an external DA service, reported excellent support, but explained that she used her expertise to know *“where to look and what questions to ask”,* suggesting that people with *“less [DA] experience [may not] feel as supported.”* Less positively, a dentistry HR respondent[R213] sought an external HR company’s support and wrote, *“at times it felt like the [company] followed a process like robots and didn’t realise a person was undergoing the ordeal.”*

### What might improve support?

Respondents wanted a more formalised but flexible response to staff-survivors, with the options listed in Table 2 adapted for context. They wanted specific training and knowledge of organisations that might help. Two respondents (an ambulance service safeguarding lead and GP staff member) reported that they wanted specific, in-house support for staff-survivors.

A few responses emphasised the need for change in people’s attitudes and behaviours, firstly dismantling sexism and hierarchical power when the DA perpetrator was a doctor:

In a previous workplace…a [line manager for many consultants] took great pride in publicising his approach of joining with like-minded colleagues to robustly support, with statements in court, a wife-beating consultant facing prison. He avoided prison. They continue to congratulate each other and remember their power and influence (Acute trust doctor and line manager[R76]).

And secondly, creating conditions for people to express empathy and compassion for colleagues. While one GP manager indicated that they “*don’t see a cut-off [between home and work]: what’s bothering them at home will come into the workplace.* (GP line manager[R11]), another indicated a lack of empathy from others and a belief in myths that ‘someone like them’ would not experience DA:

I’m not sure that people who’ve never experienced it can ever understand it. I am a really strong person and people don’t expect someone like me to have experienced it. I receive comments like this all the time. It’s a difficult subject to fully understand (GP practice manager[R288]).

## Discussion

This article is the first to explore support offered to DA-affected healthcare colleagues and experiences of providing workplace support, focusing on staff with formal support roles, mainly in primary and community healthcare settings.

### Findings summary

Positive findings were: some workplaces had staff DA policies; support via changes to working patterns, special leave, and permission to attend appointments were offered; smaller organisations enabled more tailored and responsive support; DA workers tended to support staff; and respondents’ actions to support affected colleagues were positive.

However, most respondents did not know of a staff DA policy, or reported there was no such policy – despite a 2019^19^ recommendation that workplaces should have standalone staff DA policies. Community hospitals and services made up the highest proportion of workplaces with policies. Unexpectedly, given the presence of DA advocacy programmes in GP and dentistry, no one from these areas reported a policy. This finding contrasts with study findings we report elsewhere [25]: respondents who completed the survey as a survivor (without any support role for others), including from general practice, were aware of staff DA policies. The key finding from the current study is that those who might support others were unaware of policies. Respondents indicated that awareness might be low due to high staff turnover. They felt that good policies should be proactive and flexible, not tokenistic with a tick-box approach. Support measures that NHS Employers recommends [18] were uncommon, or respondents were unaware of them. These findings suggests that workplaces may have made little progress since the 2019 survey [19], where only 1% of healthcare workforces had staff DA policies with all the recommended support measures in place, but our study’s limitations mean further research is required.

Very few respondents reported having training on supporting affected colleagues. If respondents had undertaken training, it was mostly self-directed e-learning, undertaken by motivated individuals. Training alone may not change practice, though training coupled with a named DA worker for staff might [8]. But awareness of whether their workplace had a DA worker was low. Previous research has indicated that healthcare-based DA advocates receive staff disclosures, but no specific staff advocate roles exist [7,28]. Some workplaces had resources for staff, but not well-displayed and/or accessed, highlighting that a communications strategy for policies and resources is needed.

Just under half of respondents had received DA disclosures from colleagues. However, this question had the most missing data. Although free-text responses provided good case studies, few respondents indicated having taken even basic actions, such as signposting and validating the survivors’ experiences. Even subtly negative responses and actions can invalidate survivors, deepen shame, and deter further help-seeking [29]. Generally, respondents themselves received support from external DA services or the police.

A broader finding is that a change in attitudes and beliefs may be needed to generate genuine support for survivors. Donovan and colleagues [11] found that the myths associated with DA (particularly, who experiences it) led doctors to doubt their own experience for longer and created help-seeking barriers, such as shame and stigma. This stigma intersects with sexism embedded in medicine, which allows doctor-perpetrators to evade sanctions, as research and reports about sexual misconduct in medicine have indicated [21].

### Strengths and limitations

This research is limited by the small and self-selected sample, with substantial missing data from incomplete surveys, mostly from respondents who had already completed part 1 of the survey (which was for respondents who had personal experience of DA). Drop-out may have been due to fatigue or time (especially given their busy HCW roles and the emotionally challenging topic). The ‘save and finish later’ function was under-used for unclear reasons. Missing data (and attrition) may have biased the results given that non-completers were largely HCW-survivors: according to one study, HCW-survivors are better equipped to respond to patients who are survivors [30]. Support-seeking for oneself may increase awareness of support for others. With their data, findings may have indicated greater presence and awareness of support. At the same time, overall data are limited by selection bias: those with greater interest in DA may have been more likely to complete the survey and have awareness of support options. If the survey was mandated for all staff, awareness rates may have been much lower.

Respondents were spread across multiple disciplines. We did not ask respondents about their management of or responses to potential perpetrators, which requires dedicated investigation. The wide-reaching distribution strategy, while appropriate for our exploratory research, precluded a response rate calculation, which along with missing data, the small sample, and selection bias, severely limits the generalisability, representativeness, and interpretation of the findings. Despite these limitations, our quantitative data has allowed us to provide valuable insight into a range of approaches in practice, strengthened by the complementary qualitative data. We hope that this article inspires further research as well as reflection, audit, and evaluations within services to explore the effectiveness of potential DA support options.

### Implications for research and/or practice

Given widespread calls for HCWs to identify and respond to DA among patients and that workplaces have opportunities to be safe spaces for survivors and help them to end relationships with perpetrators [12,16], there is an imperative for workplace support for HCW staff-survivors. We recommend that health services implement support measures (suggested in policies and by survivors), staff-facing DA advocates, and that all DA training about patients attends to staff experiences and needs. We know of no international peer-reviewed research about healthcare employers’ responses to DA, constraining comparisons with other countries. However, we recommend that paid DA leave, available in some countries, be offered more widely. We also recommend comparative pan-European or global research to understand healthcare employers’ responses to affected employees to surface good practice and foster cross-learning. Research should employ targeted surveys with mechanisms to estimate reach and response rate; qualitatively investigate the topic with greater nuance and explanatory power than our largely descriptive analysis has afforded; explore appropriate responses to HCW-perpetrators; and develop, pilot, and evaluate tailored support using principles of trauma-informed practice and, for example, organisational support theory. The wraparound support provided in hospitals in Victoria, Australia could be trialled internationally, adapted for primary and community healthcare.

## Conclusion

Our study is limited including by missing data but underscores the need for robust workplace support for DA-affected HCWs. Alongside positive findings, e.g. tailored support in smaller organisations, were findings indicating gaps in provision and awareness, particularly around policies, training, and dedicated support roles. We call for further research to explore the availability and implementation of support measures pan-Europe/globally to surface good practice and foster cross-learning.

## Supplementary Material

Supplemental Material

## Data Availability

Data will be lodged with the University of Bristol Research Data Repository data.bris and assigned a unique DOI.

## Note

1 https://thewomens.r.worldssl.net/images/uploads/general-downloads/shrfv/Family_Violence_Workplace_Support_Program_Overview_04_08_21.docx.
