# Supplementary material for: Experiences of supporting primary and community healthcare workers affected by domestic abuse in the United Kingdom: A cross-sectional survey
Source: Eur J Gen Pract. 2025 Nov 10;31(1):2571600. doi: 10.1080/13814788.2025.2571600 (PMC12604119; doi:10.1080/13814788.2025.2571600)
Supplement: Supplemental Material [file IGEN_A_2571600_SM1494.zip › suppl_data/ejgp-2025-0040-File002.docx]

**Appendix 1: Safeguarding-relevant aspects of the online participant information for people completing the survey as a survivor-supporter*.**

*i.e., people who experienced domestic abuse directly and thus were eligible to complete Part 1 of the survey AND had a role supporting others and thus were eligible to complete Part 2 of the survey in addition.

**What are the possible disadvantages and risks of taking part?**

You may find it difficult or upsetting to answer these questions, but you can skip questions you feel uncomfortable or unsafe answering. You can take a break by clicking 'Save & Return Later'. We provide details of support agencies at the start and end of the survey if you feel you would like support.

**How can I complete the survey safely?**

Try to complete it in a quiet and private place where others cannot see your answers.

If you feel unsafe answering questions about abuse and control because the abusive person checks or monitors your device (i.e., your phone, tablet, or computer), do not take part in this survey on the device.

Internet browsers keep a record of all the webpages you visit, in your 'history'. The survey's webpage will show up in your history, but no one will be able to read your answers. If you feel you would be safer deleting your history, you can read about how to delete it here [website removed as it is no longer available]. You can read more about how to use the internet safely if an abusive person monitors your internet activity here [womensaid.org.uk/cover-your-tracks-online/](https://www.womensaid.org.uk/cover-your-tracks-online/)

**Will my participation in this study be kept confidential?**

This study is completely independent of the NHS and other bodies such as healthcare regulators, unions, and Royal Colleges. Your involvement will remain confidential. The only exception would be if you share your name and contact details and you disclose information that suggests a serious risk of safety to any person including yourself, or risk to a child. In these cases, the researchers will share information with relevant agencies. They would discuss this sharing with you first, if possible.

**What if I take part and then want to withdraw my data?**

You are free to withdraw your data from the research, without reason or consequence, before we conduct our final data analysis. Whether you completed all the survey questions, or whether you clicked 'Save & Return Later', please contact the researchers if you wish to withdraw. We will then permanently delete your survey response.

If you wish to withdraw your data after we have begun our final analysis, we will use the anonymised data we have collected up to that point (i.e., in reporting percentages in reports) but not your free-text responses.

Please note, to withdraw your data, we would need a way to identify your survey (for example, by your name if you stated an interest in a follow-up interview, or by you telling us one of your free-text answers).

**What if I am unsure whether I have experienced domestic abuse/ coercive control?**

Everyone's experiences are unique, and the survey questions cannot accurately determine whether you have experienced domestic abuse/coercive control. However, a domestic abuse adviser can talk with you in more detail if you feel you would like support around this: the next page lists support organisations.

**National Support Services**

We know that thinking about and supporting people who are experiencing these sorts of behaviours can be difficult.

Detailed below are national helplines and services that you can signpost staff to or seek support from yourself. They can signpost to local services.

You can print out this section. If using Windows, highlight the text, left click if using a standard mouse or touchpad, and choose 'Print Selection'. If using a Mac, highlight, then click File, Print, and select Print Selection. Or you can take a screenshot or photo of it.

In an emergency call the police on 999 (an emergency is when you, or other people, are not safe and in need of immediate help)

**Details removed for website, helplines (with opening hours), and webchats (with opening hours) for national services for

1. General domestic abuse survivors
2. LGBT+ survivors
3. Women survivors
4. Men survivors
5. Honour-based abuse survivors
6. People worried about their own behaviours
7. Employers looking to respond effectively to a disclosure of domestic abuse from an employee, or looking for other advice and guidance around supporting employees
8. Survivors who experienced abuse in childhood
9. Survivors who have experienced abuse from a child under sixteen

Details were repeated at the end of the survey.**
