# Supplementary material for: Experiences of supporting primary and community healthcare workers affected by domestic abuse in the United Kingdom: A cross-sectional survey
Source: Eur J Gen Pract. 2025 Nov 10;31(1):2571600. doi: 10.1080/13814788.2025.2571600 (PMC12604119; doi:10.1080/13814788.2025.2571600)
Supplement: Supplemental Material [file IGEN_A_2571600_SM1494.zip › suppl_data/ejgp-2025-0040-File003.docx]

**Appendix 2: Survey**

**PRESSURE: Primary and community healthcare staff as survivors of domestic abuse**

Please use 'Save & Return Later' at the bottom of the screen if you would like to take a break. [**note: this message appeared on each page**]

**Context**

1.1) What area (primary or community healthcare) do you work in?

- General Practice
- Dentistry
  - For a dental practice
  - For a dental hospital
  - Other dental [free-text]
- Optometry
  - For an eye hospital
  - For an independent optometrist or optician
  - Other optometry [free-text]
- Pharmacy
  - For a hospital pharmacy
  - For a general practice
  - For a community pharmacy
  - Other pharmacy [free-text]
- Community hospital or service
  - For a Community Health NHS Trust
  - For a community hospital within a larger trust
  - Other community [free-text]
- Other [Please tell us what area of healthcare you work in] [free-text]

1.2) Do you work for the NHS or a private practice?

- I work in a fully NHS service
- NHS England commissions my service, or part of it, and I see NHS patients, but it's a private practice (e.g., dentist, GP surgery, high street optician)
- It's fully private and I do not see NHS patients
- I'm not sure
- Other [Please tell us more, if you want to] [free-text]

2) What is your role with regards to supporting staff who have experienced domestic abuse?

- Human resources (HR)
- Occupational health
- Employee assistance programme counsellor
- Line manager to healthcare professionals
- Safeguarding lead
- Hospital IDVA
- Other [Please tell us more] [free-text]

3) Please tell us how long you have been in your current role in years and/or months (please specify whether you mean years or months). [free-text]

4) Where is the main place that you work?

- England
- Wales
- Scotland
- Northern Ireland
- Other [Please tell us where you work] [free-text]

5.1) Does your workplace have a specialist domestic abuse worker, or someone with a designated role to support patients who are experiencing domestic abuse?

- Yes

What’s their job title? (eg, Independent Domestic Violence Advisor (IDVA), advocate-educator, domestic abuse nurse, safeguarding lead) [free text]

Does this person also support staff who are experiencing domestic abuse?

- - Yes, supporting staff is part of their role
  - Yes, but I don't think supporting staff is officially part of their role
  - No, they do not support staff
  - Unsure
- No
- I don't know

5.2) Aside from people in your role, who else might support staff who are experiencing domestic abuse, e.g., HR, occupational health, staff well-being service? Please list them here. [free-text]

**Training on Supporting Staff**

6.1) Have you had any training on supporting STAFF who have experienced domestic abuse?

- No
- Yes, specific training
- Yes, within broader training e.g., staff wellbeing

6.2) [If yes] Is the training about supporting STAFF who have experienced domestic abuse repeated?

- No, it is a one-off
- Yes, it is repeated every year
- Yes, it is repeated every 3 years
- Other [Please tell us more about this, if you want to] [free-text]

6.3) What was the format of the training on supporting STAFF who have experienced domestic abuse? (Please tick all that apply)

- Self-directed e-learning
  - Who produced this training? [free-text]
  - How many hours was this training learning? (If the training is repeated each year, please tell us how many hours are in one year's training)
- In-person live training
  - Who produced this training? [free-text]
  - How many hours was this training learning? (If the training is repeated each year, please tell us how many hours are in one year's training)
- Online live training
  - Who produced this training? [free-text]
  - How many hours was this training learning? (If the training is repeated each year, please tell us how many hours are in one year's training)
- Other
- Please tell us the format, who delivered the training, and how many hours it was

**Resources to Support Staff**

7) Are resource materials about domestic abuse (e.g., posters, leaflets, etc.) available in your workplace?

- Yes, well-displayed, and accessed by staff
- Yes, well-displayed, but not accessed by staff
- Yes, but not well-displayed
- No
- Unsure
- Other [Please tell us more about this, if you want to] [free-text]

8.1) Does your workplace have a policy for STAFF who have experienced domestic abuse?

- Yes
- No
- I don't know
- Please could you upload the policy here:
- Or, you could give us a link to the policy:

Please note: we will be unable to access the link if it's on your intranet. We can only access public-facing links. Uploading or linking to your policy will mean we know which organisation you are from. If you are unable to upload or link to your policy, or do not wish to make clear your organisation please email the policy to [researchers’ email addresses removed]

8.2) [if yes] Do you think that most staff know about the policy for STAFF who have experienced domestic abuse?

- Yes
- No
- Some staff do, and some staff don't
- I don't know

Please tell us more about this if you want to [free-text]

9) Do you use any other policy or guidance, e.g., from a national body, to support STAFF who have experienced domestic abuse?

- Yes
  - What is the policy or guidance that you use?
  - Please could you upload the policy here:
  - Or, you could give us a link to the policy or guidance

Please note: we will be unable to access the link if it's on your intranet. We can only access public-facing links. If you are unable to upload or link to it please email it to [researchers’ email addresses removed]

- No
- I don't know

**Support Available in Your Workplace**

10) In your workplace, what support is there for STAFF who have experienced domestic abuse? [Please tick all that apply]

- I don't know what's available

**Working hours and duties**

- Changes to working times, days, or patterns
- Changes to specific duties (e.g., to avoid contact with an abusive person)
- Waiving the use of informal and formal stages of sickness absence management, when any sickness might be
- linked to domestic abuse
- The option for redeployment or relocation

**Leave/appointments**

- Special leave provisions (e.g., using existing leave, or the option for unpaid leave)
- Paid leave for domestic abuse
- Permission to attend appointments related to domestic abuse during work hours
- Permission to use private spaces at work to hold appointments related to domestic abuse

**Safety planning**

- Measures to ensure safety at work (e.g., blocking emails, screening phone calls, reception and security being alerted that the abusive person/people might come to the workplace)
- Measures to ensure safety while travelling to and from work
- Review of the personal information that the workplace holds on the person experiencing abuse, e.g., address
- The option to stay at work for safety (e.g., to stay late or to sleep at work)
- Permission to use work phones and computers to look up information and access support
- Training for security and reception staff about how to handle situations where an abusive person turns up at a workplace

**Referrals and signposting**

- Signposting to an in-house Independent Domestic Violence Advisor (IDVA) or domestic abuse advocate
- Referral to an Employee Assistance Programme
- Referral to Occupational Health
- Support from qualified professionals (e.g., staff counsellors or therapists)

**Pay**

- Referral to a credit union or financial advisory service
- Changes to pay arrangements

**Confidentiality**

- The right to have their disclosure kept confidential

11) Does your workplace have anything in place to manage staff who have been (or are suspected of having been) abusive or coercively controlling towards partners, ex-partners, or adult family members?

- Yes [Please tell us what your workplace has in place] [free-text]
- No

12) What do you think about your workplace/organisational response to STAFF who have experienced or used domestic abuse? [free-text]

13) What might improve your workplace/organisation's response to STAFF who have experienced or used domestic abuse?

You may find it helpful to look at the items listed towards the start of this page to help you think about your answer. [free-text]

**Domestic Abuse Among Staff**

14.1) In the past five years, have you encountered anyone experiencing domestic abuse among the STAFF members for whom you have a responsibility?

- Yes
  - How many staff members? Please tell us a number [free-text]
  - What actions, if any, did you take? In the interests of confidentiality, please do not share any names or other identifying information [free-text]
- No

14.2) [if yes] Did you get any support for yourself when responding to the staff members in question? [Tick all that apply]

- I got support from HR
- I got support from an Organisational Development business partner
- I got support from safeguarding teams in my workplace
- I got support from an IDVA or domestic abuse advocate in my workplace
- I got support from the Employee Assistance Programme
- I got support from the police
- I got support from a domestic abuse service
- I got support from elsewhere [Please tell us where you got support from, if you want to] [free-text]
- I can't remember if I got support when responding to the staff members in question
- I did not get any support when responding to the staff members in question

14.3) What did you think of the support you got for yourself when responding to the staff members in question? [free-text]

15) Has COVID-19 affected your response to STAFF who have experienced domestic abuse in any way?

- Yes [Please tell us more about this, if you want to] [free-text]
- No

**About You & Opportunity for a Follow-up Interview**

This is the end of our questions about domestic abuse. If there are other things you would like to tell us, please do so [free-text]

The final few questions ask for information about you. We ask about this 'special category personal data' to help ensure we capture experiences from a range of people. You can skip any questions you do not want to answer.

16) Age

- 18-25
- 26-35
- 36-45
- 46-55
- 56-65
- 66+
- Prefer not to say

17) Are you...

- Male
- Female
- Non-binary/genderqueer
- Intersex
- Another term [Please tell us the term, if you want to] [free-text]

18) Are you transgender?

- Yes
- No
- Not sure

19) What is your ethnicity?

- White: English/Welsh/Scottish/Northern Irish/British
- White Irish
- Gypsy or Irish Traveller
- Other White
- White and Black Caribbean
- White and Black African
- White and Asian
- Other Mixed
- Indian
- Pakistani
- Bangladeshi
- Chinese
- Other Asian
- Black African
- Black Caribbean
- Other Black
- Arab
- Other [Please tell us your ethnicity, if you want to] [free-text]
- Prefer not to say

20) Do you have a religion?

- No religion
- Buddhist
- Christian
- Hindu
- Jewish
- Muslim
- Sikh
- Other [Please tell us your religion, if you want to] [free-text]

21) Do you have parental responsibility for children under 18?

- Yes
- No
- Prefer not to say

22) What is your sexual orientation?

- Heterosexual
- Bisexual
- Gay/Lesbian
- Pansexual
- Other [Please tell us your sexual orientation, if you want to] [free-text]
- Prefer not to say

23) Do you consider yourself to have a disability, or to be Disabled? (We use both disability and Disabled here, because different people prefer different terms)

- Yes [Please tell us more about this, if you want to] [free-text]
- No
- Prefer not to say

**Thank you so much for taking part in this survey. We really value the time you have taken, and we appreciate that we have asked some difficult questions.**

**24) Would you be interested in taking part in a follow-up interview? If you say yes now, but change your mind later, that is completely fine.**

- Yes [respondent is taken to a separate form for contact details]
- No
