# Supplementary material for: Experiences of supporting primary and community healthcare workers affected by domestic abuse in the United Kingdom: A cross-sectional survey
Source: Eur J Gen Pract. 2025 Nov 10;31(1):2571600. doi: 10.1080/13814788.2025.2571600 (PMC12604119; doi:10.1080/13814788.2025.2571600)
Supplement: Supplemental Material [file IGEN_A_2571600_SM1494.zip › suppl_data/ejgp-2025-0040-File004.docx]

**Appendix 3: Questions (Q) with missing data**

The number of records missing goes up as the survey progresses. Data were missing for questions on

- Q5.1: Domestic abuse worker: n=15 missing (n=13 were ‘survivor-supporters’*)
- Q6.1: Training: n=17 missing (n=13 were survivor-supporters)
- Q7: Availability of resources: n=18 missing (n=13 were survivor-supporters)
- Q8.1: Presence of a staff domestic abuse policy: n=18 missing (the same as Q7).
- Q14.1: Experience of providing support: n=28 missing (the same as Q7 plus 10 more; overall n=23 were survivor-supporters).

*denotes people who experienced domestic abuse directly (and thus were eligible to complete Part 1 of the survey) AND had a role supporting others (and thus were eligible to complete Part 2 of the survey in addition).
