# Supplementary material for: Experiences of supporting primary and community healthcare workers affected by domestic abuse in the United Kingdom: A cross-sectional survey
Source: Eur J Gen Pract. 2025 Nov 10;31(1):2571600. doi: 10.1080/13814788.2025.2571600 (PMC12604119; doi:10.1080/13814788.2025.2571600)
Supplement: Supplemental Material [file IGEN_A_2571600_SM1494.zip › suppl_data/ejgp-2025-0040-File005.docx]

**Appendix 4: Cross-tabulation of results by general practice, dentistry, community healthcare, and other**

**Table A4.1 Support measures**

| **Support measures** | **General practice** | **Dentistry** | | | **Community healthcare** | | | **Other** | | | **Total** | | |
| --- | --- | --- | --- | --- | --- | --- | --- | --- | --- | --- | --- | --- | --- |
| The right to confidentiality | 4 | 3 | | | 4 | | | 7 | | | 18 | | |
| **Working patterns** |  | | |  | | |  | | |  | | |  |
| Changes to working times, days, or patterns | 2 | 2 | | | 3 | | | 5 | | | **12** | | |
| Changes to specific duties (e.g., to avoid contact with perpetrators) | 2 | 2 | | | 1 | | | 4 | | | **9** | | |
| Option for redeployment or relocation | - | 1 | | | 2 | | | 3 | | | **6** | | |
| Waiving the use of informal and formal sickness absence management stages when sickness might be domestic abuse-linked | 1 | 2 | | | - | | | 1 | | | **4** | | |
| **Leave** |  | | |  | | |  | | |  | | |  |
| Special leave provisions (e.g., using existing leave, or the option for unpaid leave) | 3 | 2 | | | 2 | | | 5 | | | **12** | | |
| Permission to attend appointments related to domestic abuse during work hours | 3 | 3 | | | 2 | | | 4 | | | **12** | | |
| Permission to use private spaces at work to hold domestic abuse-related appointments | 1 | 3 | | | 1 | | | 2 | | | **7** | | |
| Paid leave for domestic abuse | 1 | 1 | | | 1 | | | 1 | | | **4** | | |
| **Safety planning** |  | | |  | | |  | | |  | | |  |
| Workplace safety measures e.g., blocking emails, screening phone calls, -reception & security alerted that perpetrator might come to the workplace | 4 | | - | | | 2 | | | 3 | | | **9** | |
| Permission to use work phones/computers to look up information and access support | 3 | | 3 | | | 1 | | | 2 | | | **9** | |
| Review of personal information held by workplace e.g., address | 2 | | - | | | 1 | | | 3 | | | **6** | |
| Measures to ensure safety while travelling to and from work | 2 | | - | | | 2 | | | 1 | | | **5** | |
| Option to stay at work for safety (e.g., to stay late or to sleep at work) | 3 | | - | | | 1 | | | 1 | | | **5** | |
| Training for security and reception staff for managing perpetrator who turns up at workplace | 3 | | - | | | 1 | | | 1 | | | **5** | |
| **Referrals** |  | | |  | | |  | | |  | | |  |
| Occupational health referral | 3 | | 1 | | | 3 | | | 4 | | | **11** | |
| Support from qualified professionals (e.g., staff counsellors or therapists) | 3 | | - | | | 3 | | | 4 | | | **10** | |
| Referral to an employee assistance program | - | | 1 | | | 1 | | | 4 | | | **6** | |
| Signposting to an in-house domestic abuse advocate | - | | - | | | 1 | | | 4 | | | **5** | |
| **Pay** |  | |  | | |  | | |  | | |  | |
| Changes to pay arrangements | 2 | | 1 | | | 2 | | | 2 | | | **7** | |
| Referral to a credit union or financial advisory service | 2 | | - | | | 1 | | | 1 | | | **4** | |

**Table A4.2: Training on supporting staff-survivors**

|  | **Relevant training within broader training** |
| --- | --- |
| General Practice | 3 |
| Dentistry | 3 |
| Community healthcare | 3 |
| Other | - |
| **TOTAL** | **9** |

**Table A4.3 Frequency of training**

|  | **Relevant training one-off** | **Repeated annually** | **Repeated every 3 years** |
| --- | --- | --- | --- |
| General Practice | 1 | - | 1 |
| Dentistry | 2 | 1 | 1 |
| Community healthcare | 1 | - | 1 |
| Other | - | 1 | - |
| **TOTAL** | **4** | **2** | **3** |

**Table A4.4 Format of training (including n=2 who had specific training)**

|  | **E-learning** | **Online live** | **In-person live** | **Unspecified** |
| --- | --- | --- | --- | --- |
| General Practice | 2 | 1 |  |  |
| Dentistry | 2 | 1 |  |  |
| Community healthcare | 1 | 1 |  |  |
| Other | - | 1 | 1 | 1 |
| **TOTAL** | **5** | **4** | **1** | **1** |

**Table A4.5 Is there a specialist domestic abuse worker to support patients?**

|  | **Yes** | **No** | **I don’t know** |
| --- | --- | --- | --- |
| General Practice | 5 | 5 | 1 |
| Dentistry | 2 | 6 | 1 |
| Community | 7 | 3 | 5 |
| Other | 4 | 4 | 4 |
| **TOTAL** | **18** | **18** | **11** |

**Table A4.6 Do they also support staff?**

|  | **Yes** | **Yes, but not as an official part of their role** | **No** | **Unsure** |
| --- | --- | --- | --- | --- |
| General Practice | - | 2 | 2 | 1 |
| Dentistry | 2 | - | - | 1 |
| Community | 3 | 3 | - | 1 |
| Other | 1 | 1 | 1 | - |
| **TOTAL** | **6** | **6** | **3** | **3** |

**Table A4.7 Are there resource materials such as posters about domestic abuse in the workplace?**

|  | **Yes, well displayed and accessed by staff** | **Yes, but not well-displayed** | **Yes, well-displayed, but not accessed by staff** | **No** | **Unsure** | **Other** |
| --- | --- | --- | --- | --- | --- | --- |
| General Practice | 2 | 1 | - | 1 | 4 | 2 |
| Dentistry | 0 | 1 | - | - | - | 2 |
| Community | 8 | 1 | 1 | 5 | 4 | - |
| Other | 2 | 3 | 1 | 4 | 2 | - |
| **TOTAL** | **12** | **6** | **2** | **10** | **10** | **4** |

**Table A4.8 Experiences of providing support (encountered affected staff within past 5 years)**

|  | **Yes** | **No** |
| --- | --- | --- |
| General Practice | 4 | 6 |
| Dentistry | 4 | 1 |
| Community | 5 | 6 |
| Other | 3 | 5 |
| **TOTAL** | **16** | **18** |

**Table A4.9 Support to support others**

|  | **From** | | | | | | | | |
| --- | --- | --- | --- | --- | --- | --- | --- | --- | --- |
|  | **External domestic abuse service** | **Safeguarding** | **Police** | **Human resources** | **Employee assistance programme** | **Organisational development business partner** | **Healthcare based domestic abuse worker** | **External human resources** | **Elsewhere** |
| General Practice | 2 | 1 | 1 | 1 | 1 | 1 | - | - | - |
| Dentistry | - | - | 1 | 1 | 1 | - | - | 1 | 1 |
| Community | 2 | 3 | 1 | 1 | 1 | - | 1 | - | 1 |
| Other | 1 | 1 | 1 | 1 | - | - | - | - | 1 |
| **TOTAL** | **5** | **5** | **4** | **4** | **3** | **1** | **1** | **1** | **3** |
