# Supplementary material for: Experiences of supporting primary and community healthcare workers affected by domestic abuse in the United Kingdom: A cross-sectional survey
Source: Eur J Gen Pract. 2025 Nov 10;31(1):2571600. doi: 10.1080/13814788.2025.2571600 (PMC12604119; doi:10.1080/13814788.2025.2571600)
Supplement: Supplemental Material [file IGEN_A_2571600_SM1494.zip › suppl_data/tejp-2025-0043-File025.pdf]

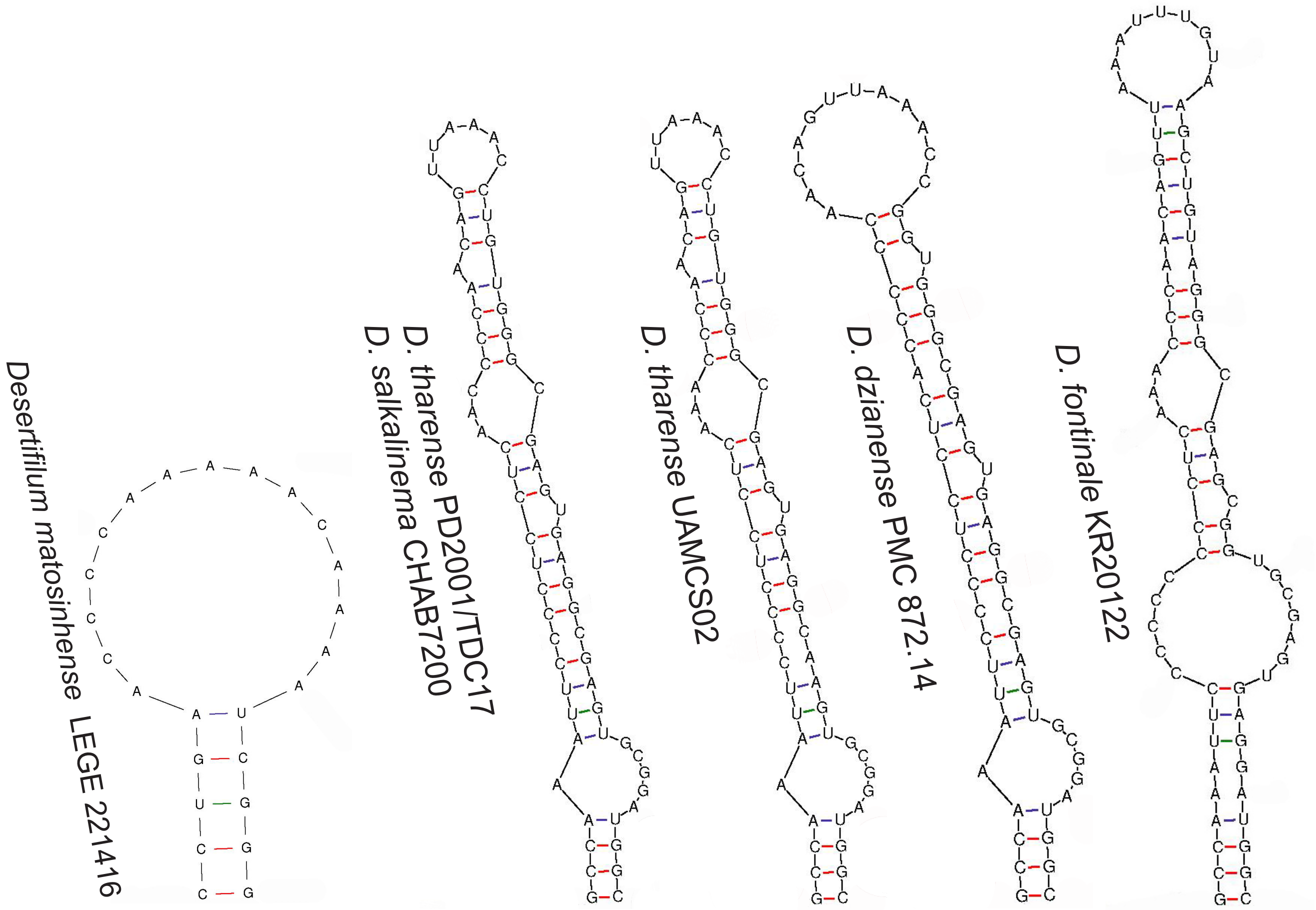
Desertifilum matosinhense LEGE 221416
D. tharense PD2001/TDC17
D. salkalinema CHAB7200
D. tharense UAMCS02
D. dzianense PMC 872.14
D. fontinale KR20122
