# Supplementary figures and images for: Experiences of supporting primary and community healthcare workers affected by domestic abuse in the United Kingdom: A cross-sectional survey
Source: Eur J Gen Pract. 2025 Nov 10;31(1):2571600. doi: 10.1080/13814788.2025.2571600 (PMC12604119; doi:10.1080/13814788.2025.2571600)

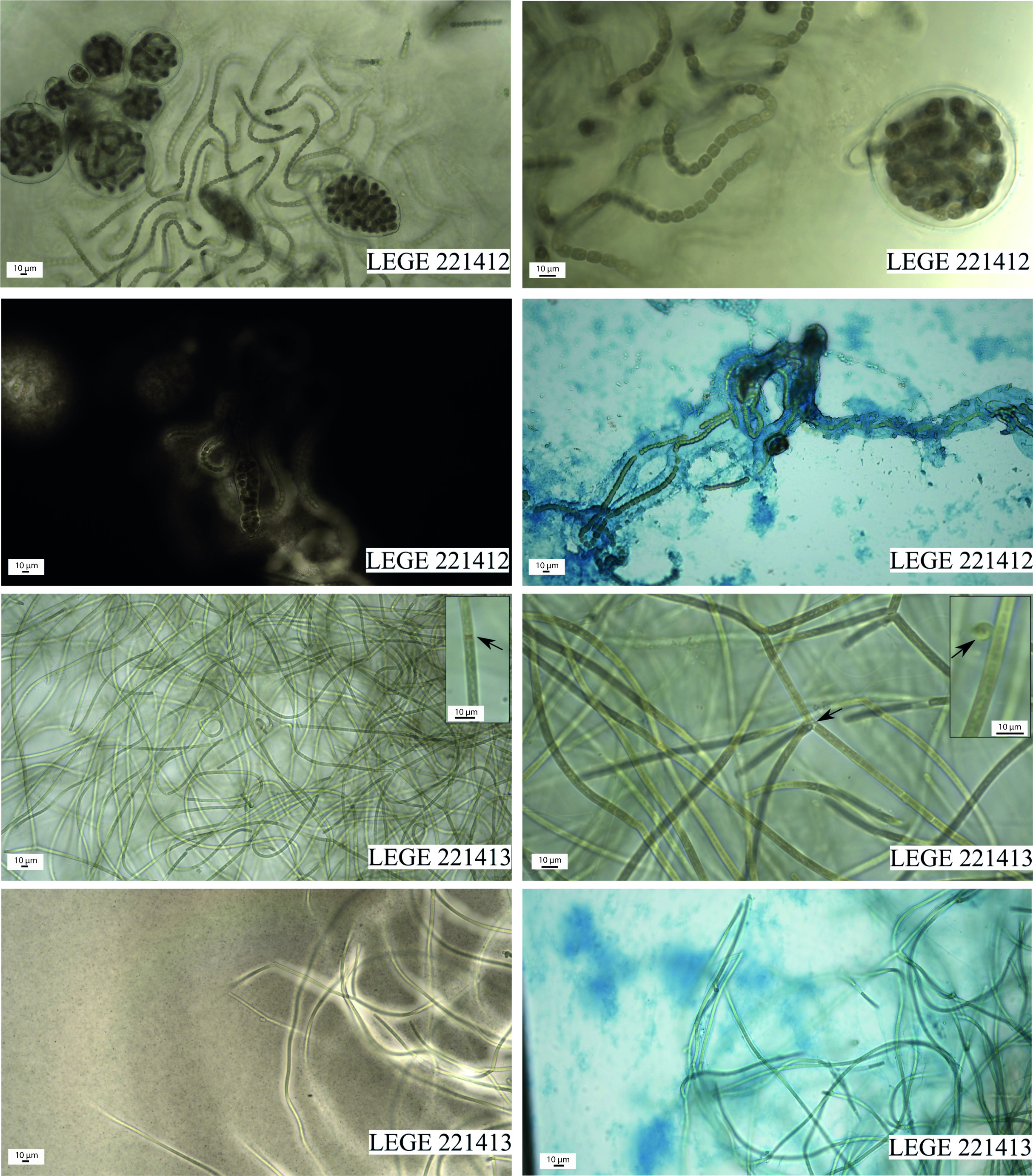

Supplement: Supplemental Material [file IGEN_A_2571600_SM1494.zip › suppl_data/tejp-2025-0043-File014.jpg]

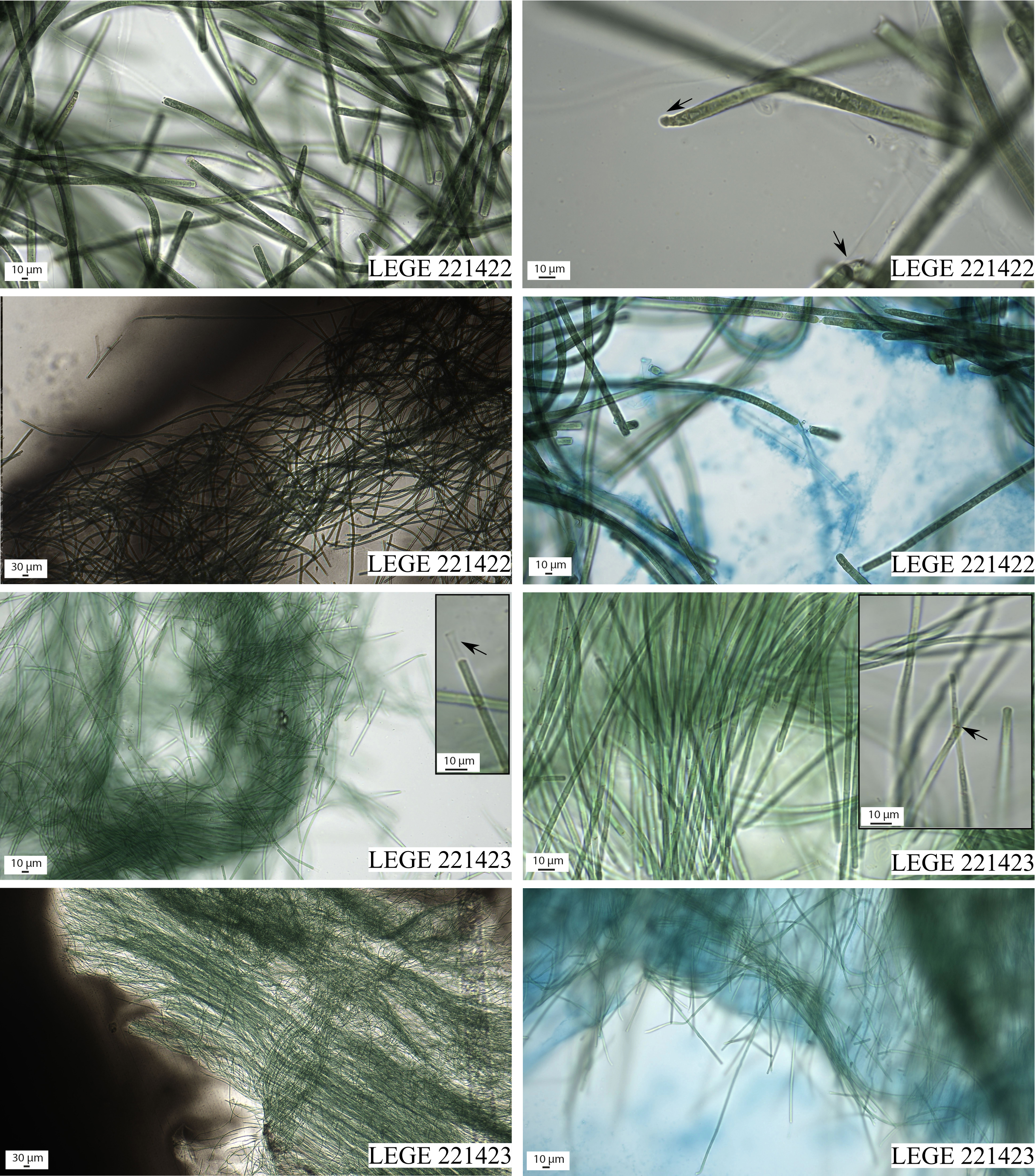

Supplement: Supplemental Material [file IGEN_A_2571600_SM1494.zip › suppl_data/tejp-2025-0043-File015.jpg]

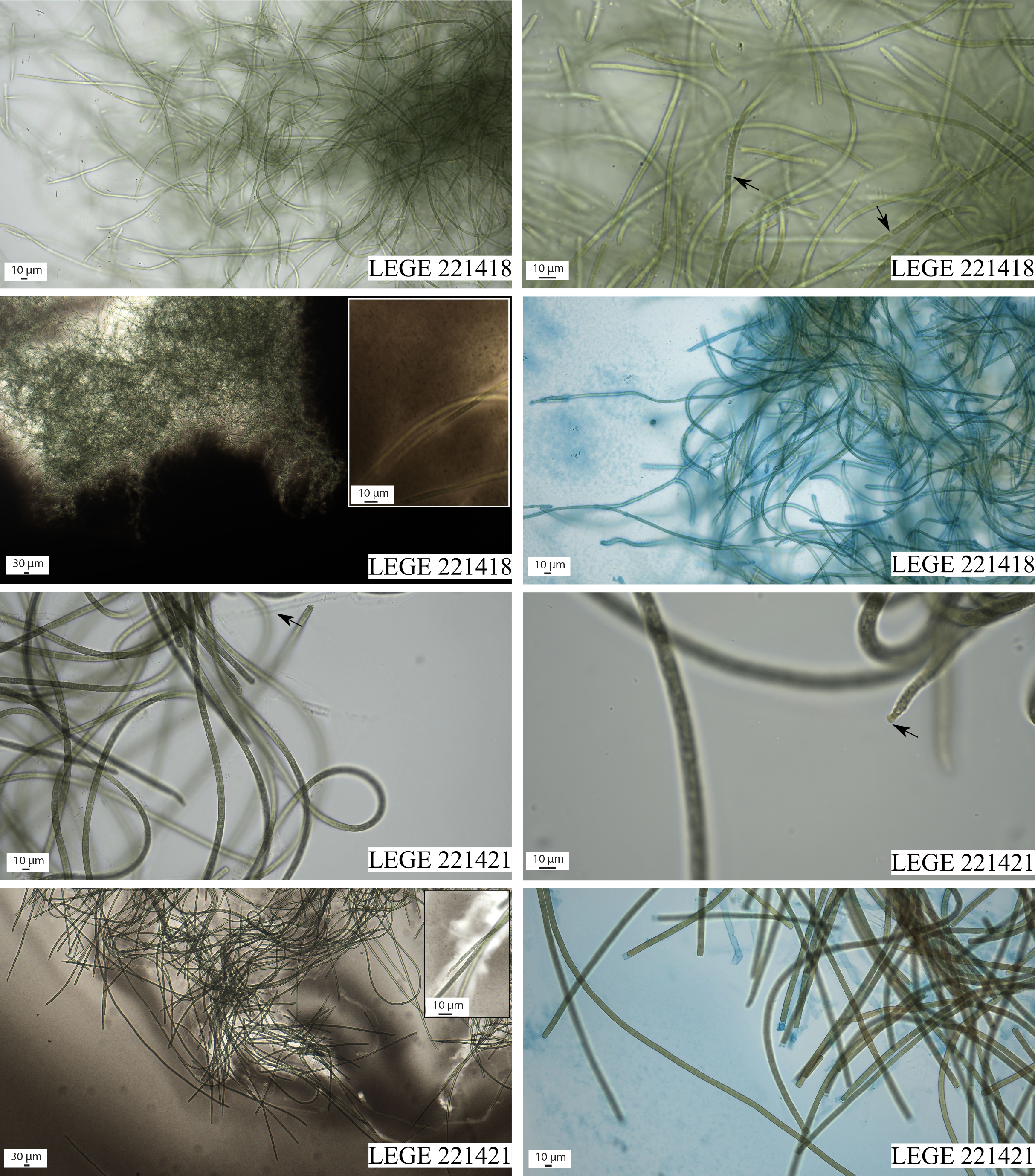

Supplement: Supplemental Material [file IGEN_A_2571600_SM1494.zip › suppl_data/tejp-2025-0043-File016.jpg]

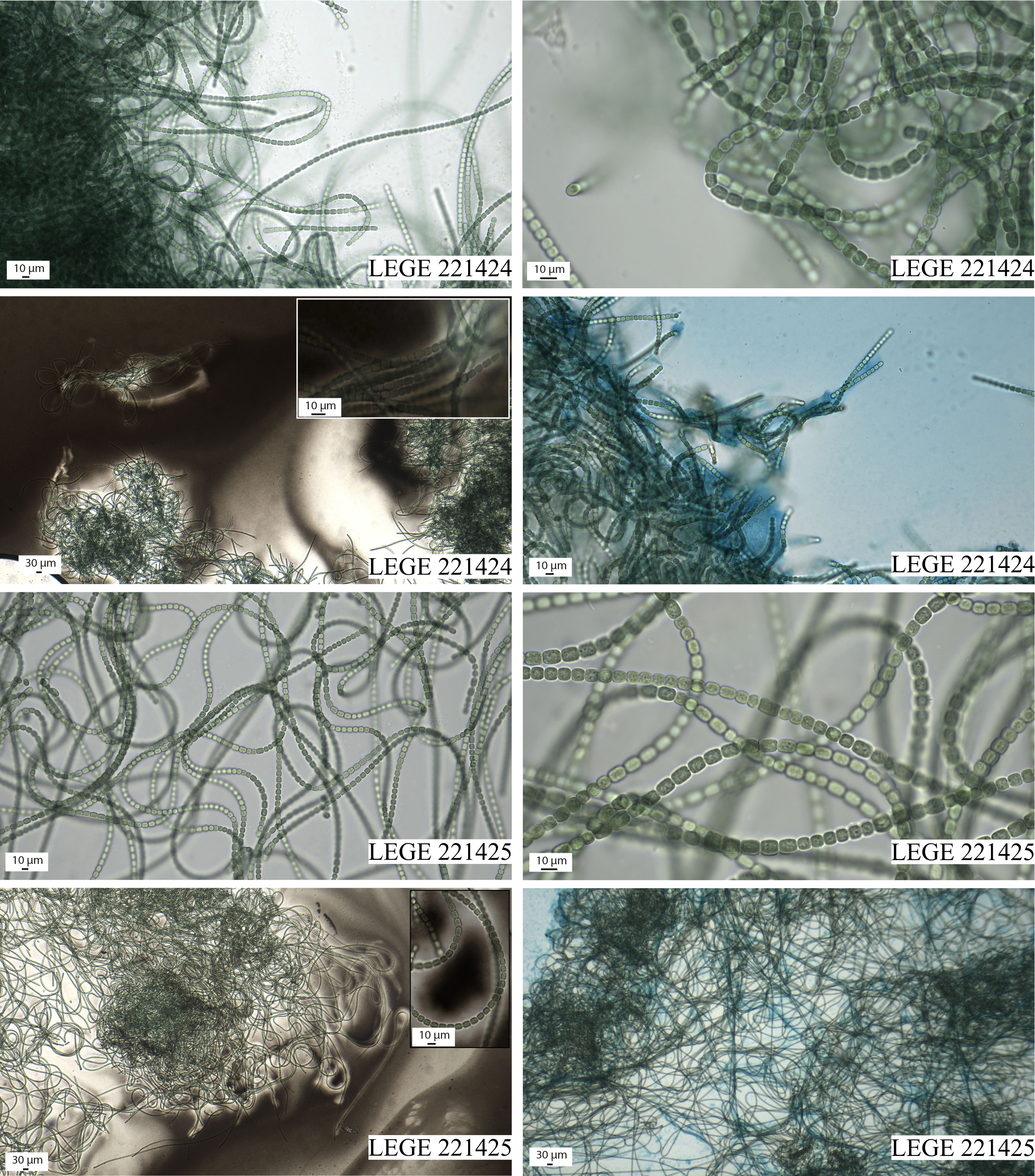

Supplement: Supplemental Material [file IGEN_A_2571600_SM1494.zip › suppl_data/tejp-2025-0043-File017.jpg]

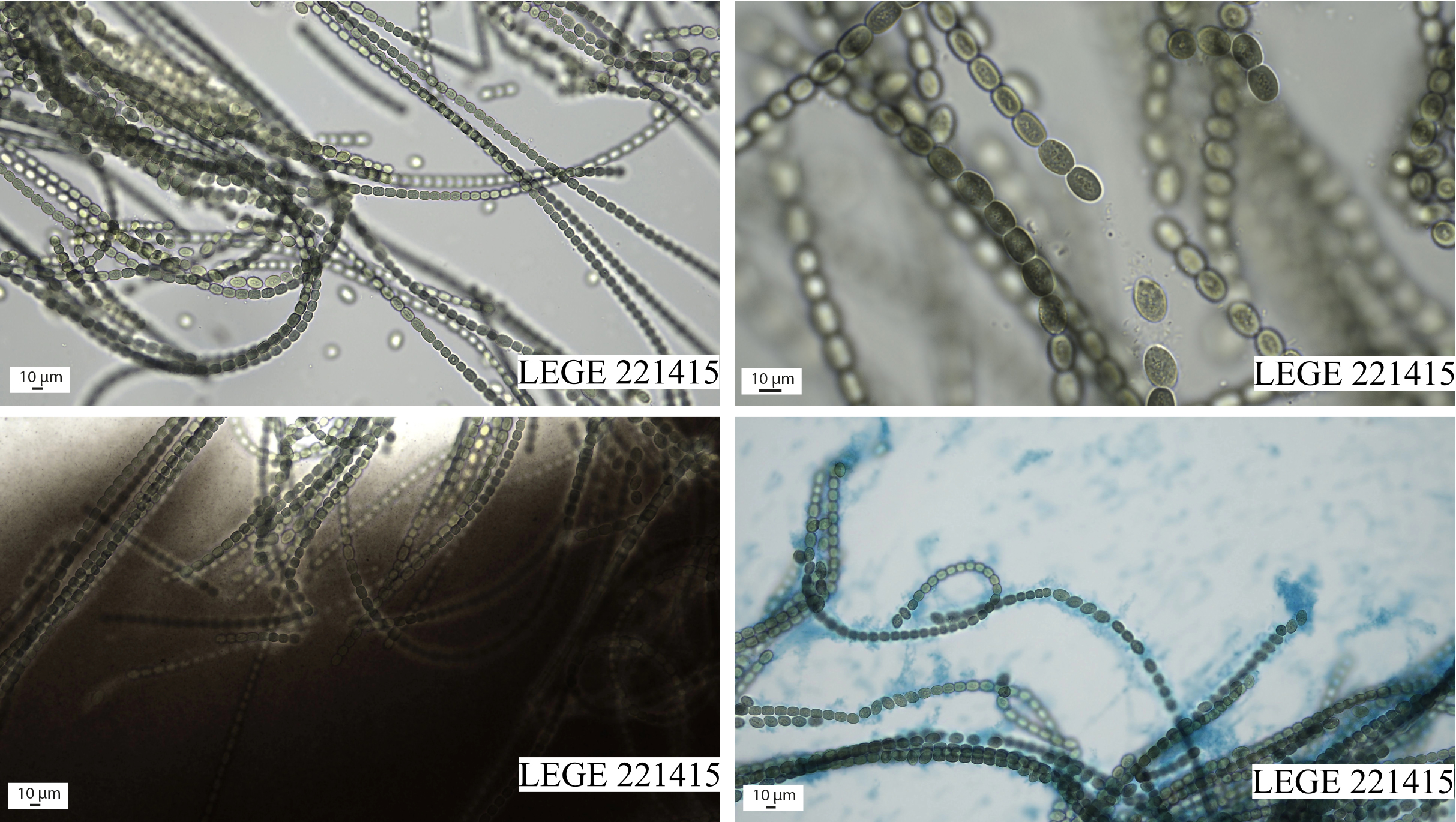

Supplement: Supplemental Material [file IGEN_A_2571600_SM1494.zip › suppl_data/tejp-2025-0043-File018.jpg]

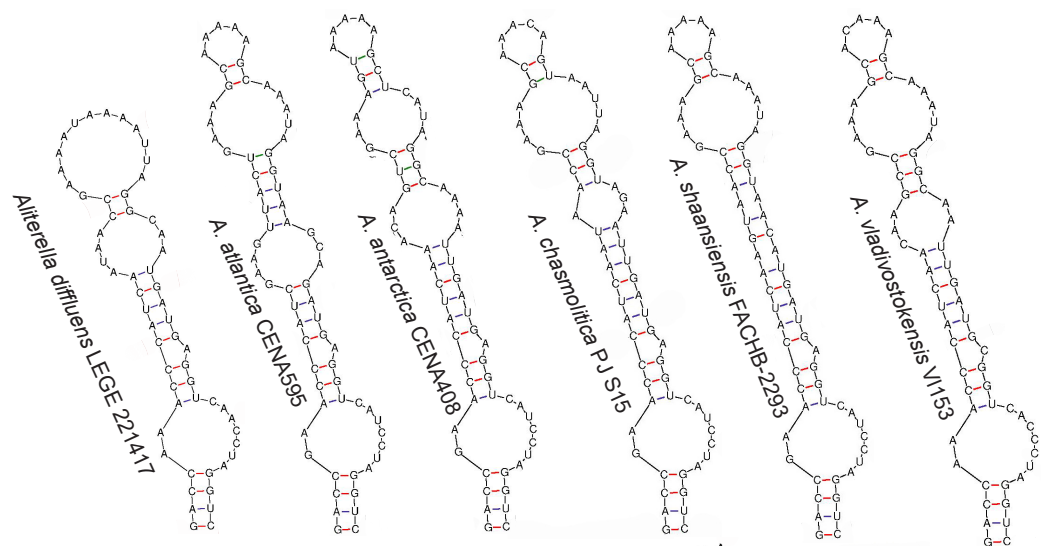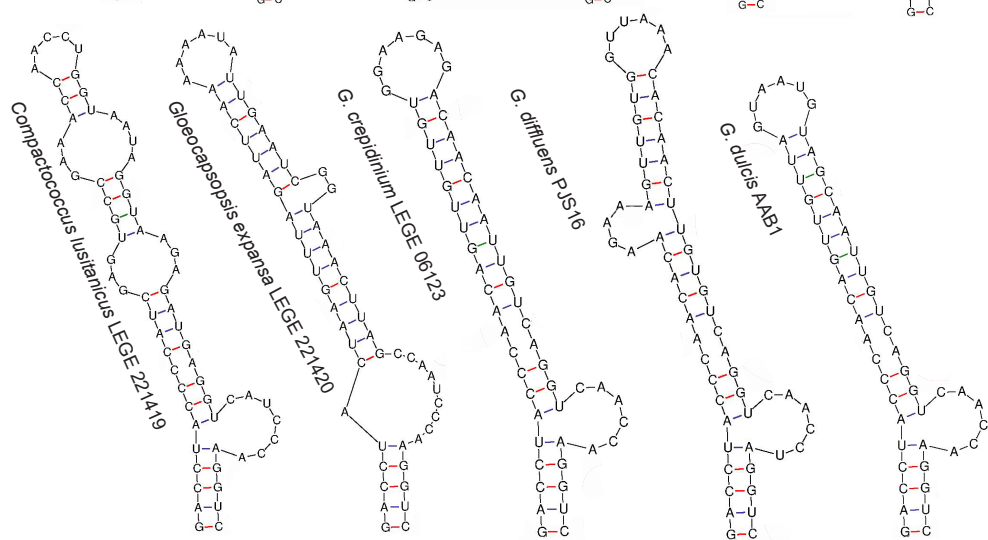

Supplement: Supplemental Material [file IGEN_A_2571600_SM1494.zip › suppl_data/tejp-2025-0043-File019.pdf]

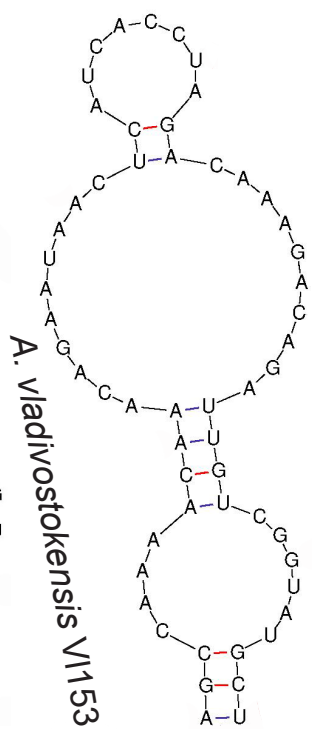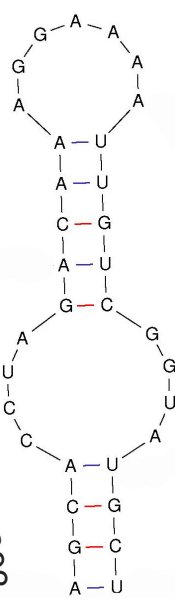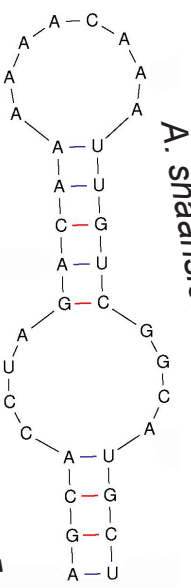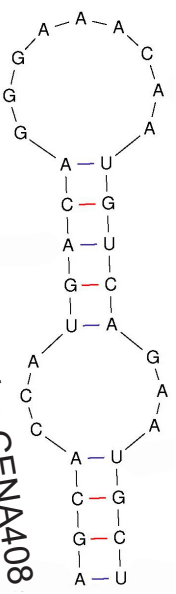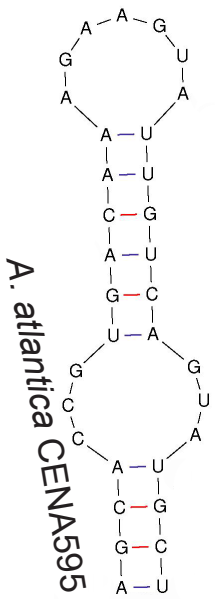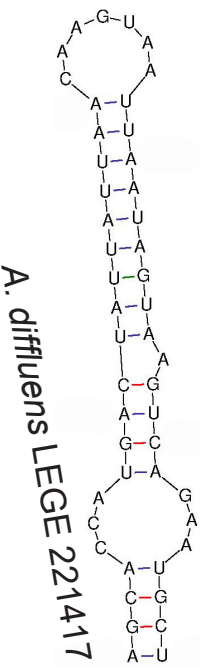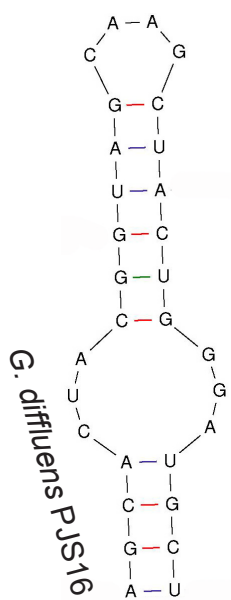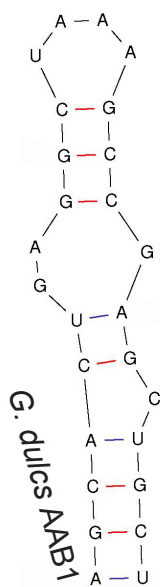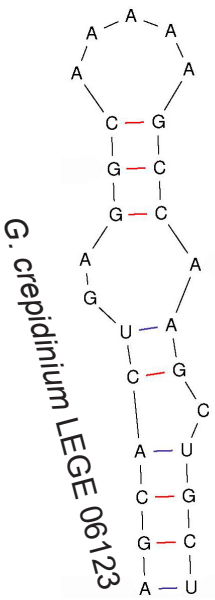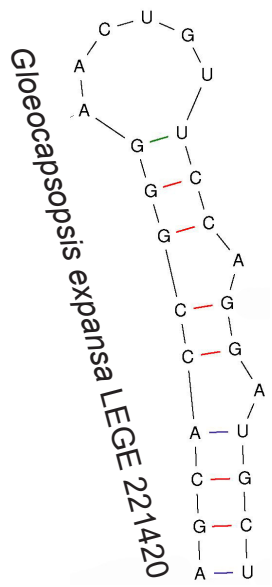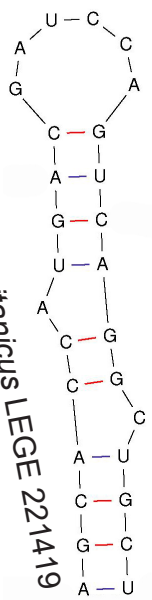

Supplement: Supplemental Material [file IGEN_A_2571600_SM1494.zip › suppl_data/tejp-2025-0043-File020.pdf]

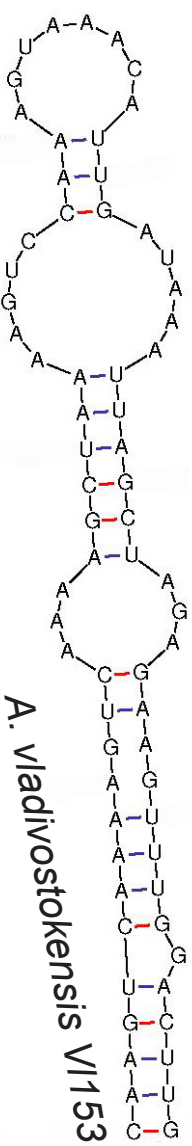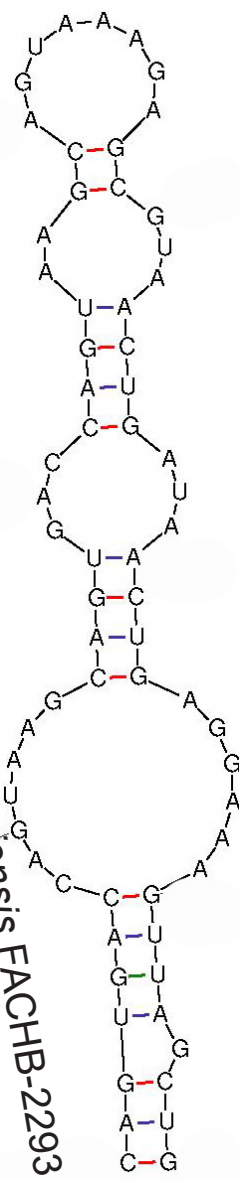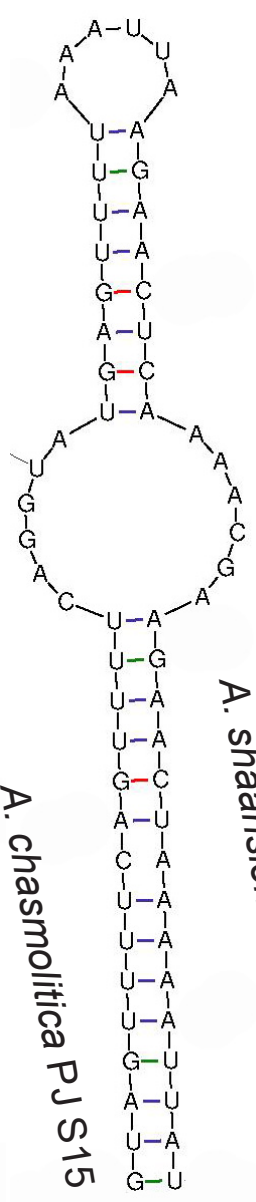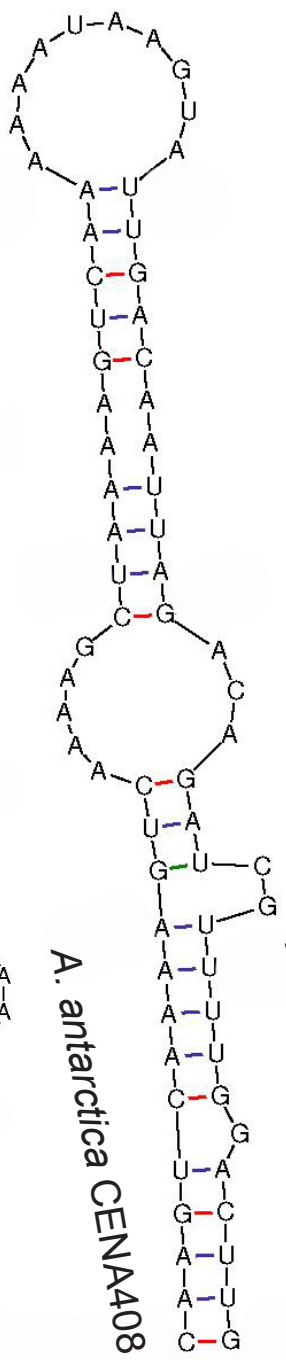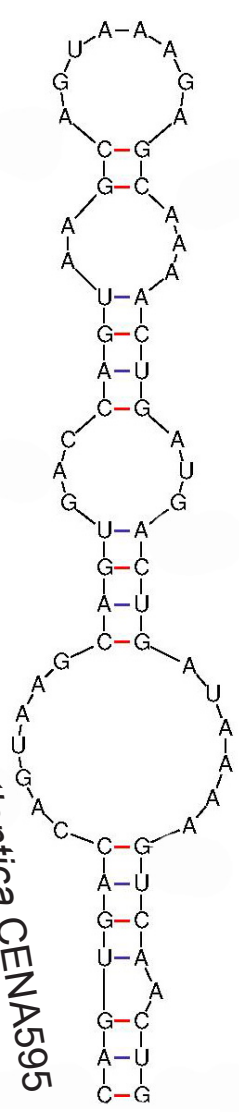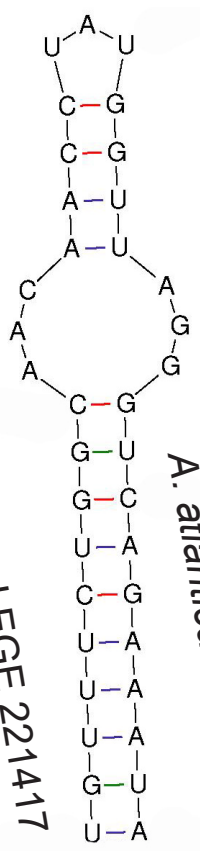

Supplement: Supplemental Material [file IGEN_A_2571600_SM1494.zip › suppl_data/tejp-2025-0043-File021.pdf]

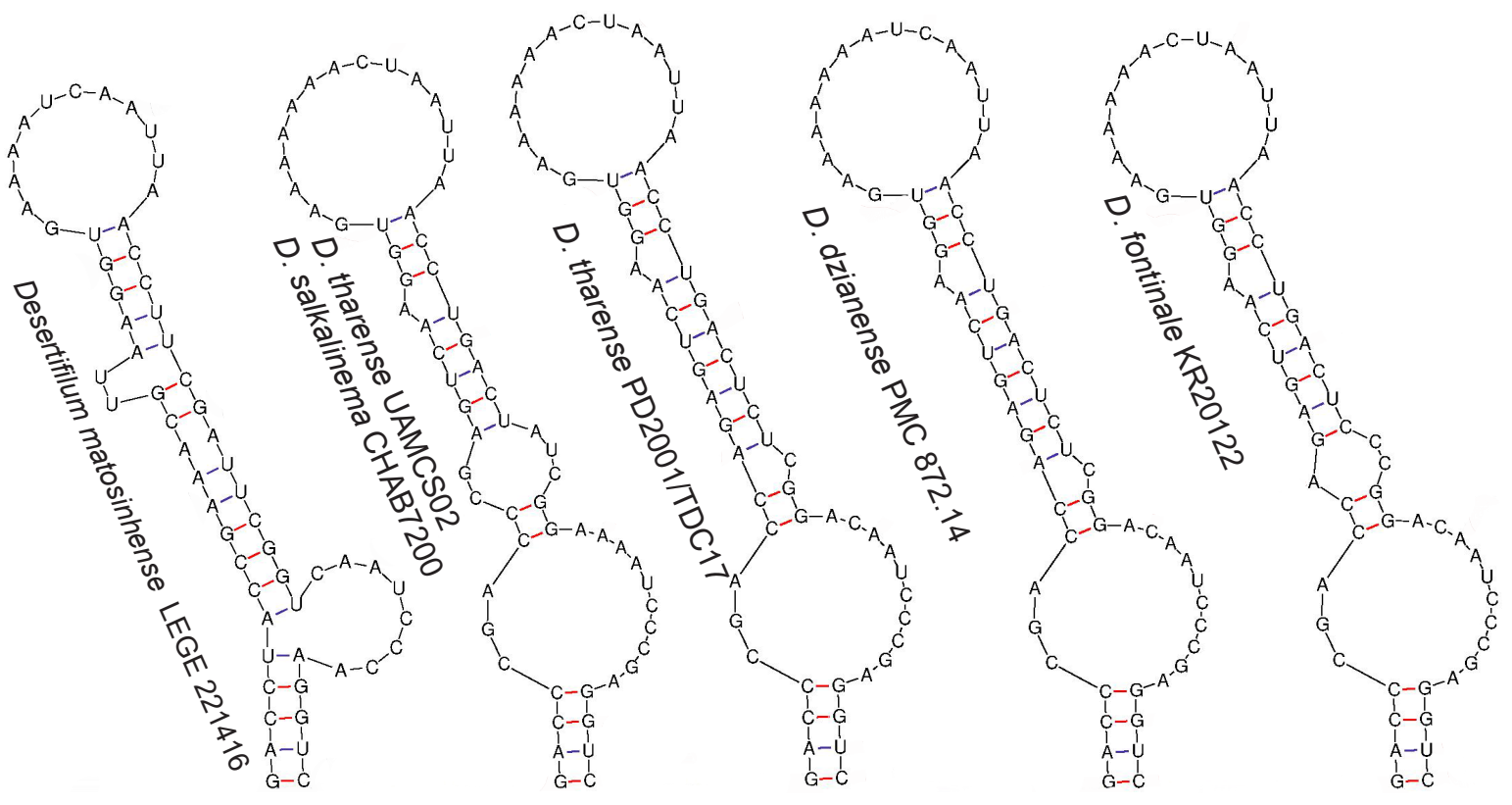

Supplement: Supplemental Material [file IGEN_A_2571600_SM1494.zip › suppl_data/tejp-2025-0043-File023.pdf]

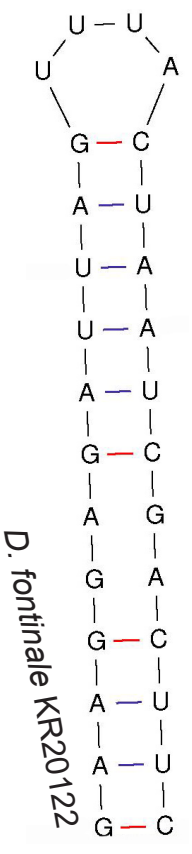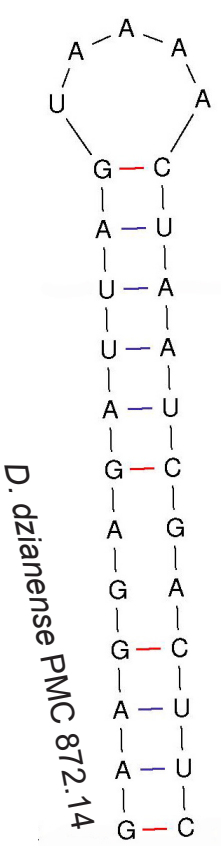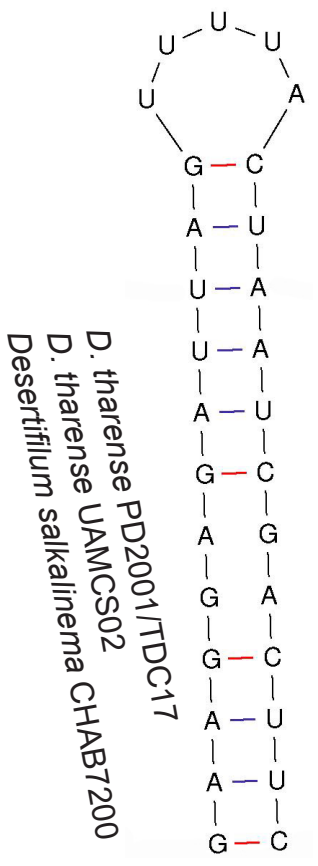

*D. tharense* UAMCS02  
*D. tharense* CHAB7200  
*Desertifilum salkalinema*

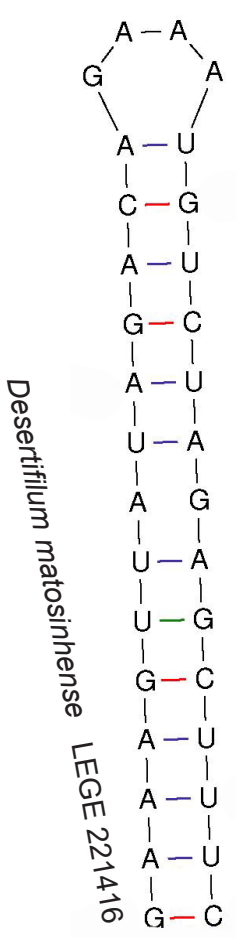

Supplement: Supplemental Material [file IGEN_A_2571600_SM1494.zip › suppl_data/tejp-2025-0043-File026.pdf]

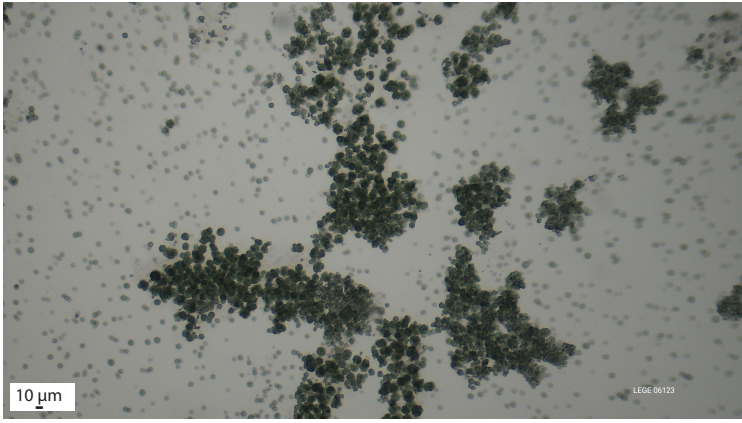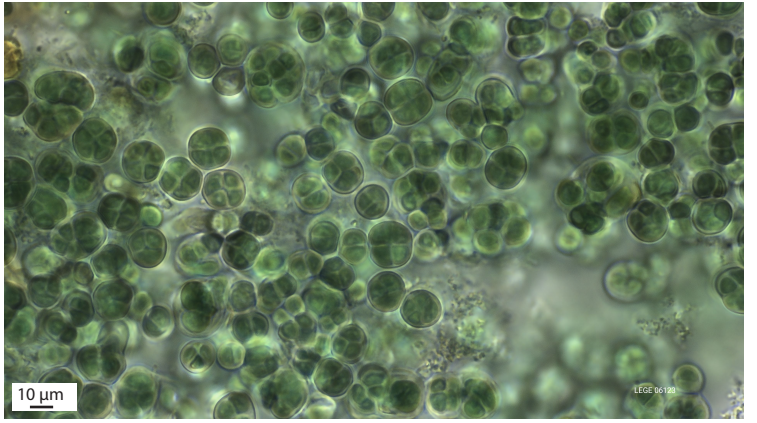

Supplement: Supplemental Material [file IGEN_A_2571600_SM1494.zip › suppl_data/tejp-2025-0043-File027.pdf]
