# Supplementary material for: Experiences of supporting primary and community healthcare workers affected by domestic abuse in the United Kingdom: A cross-sectional survey
Source: Eur J Gen Pract. 2025 Nov 10;31(1):2571600. doi: 10.1080/13814788.2025.2571600 (PMC12604119; doi:10.1080/13814788.2025.2571600)
Supplement: Supplemental Material [file tejp-2025-0043-File013.pdf › suppl_data/tejp-2025-0043-File013.pdf]

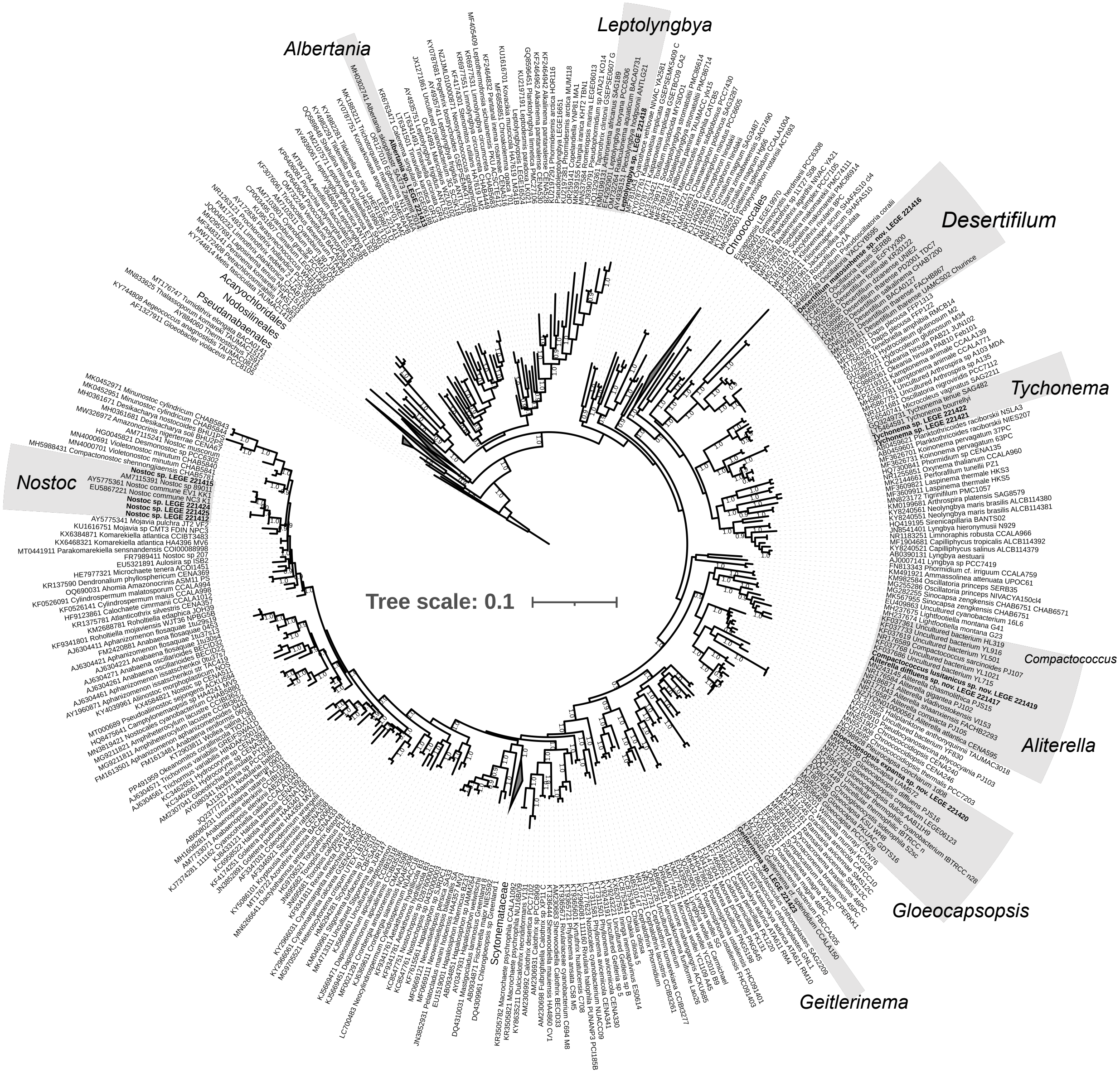
Albertania
Leptolyngbya
Desertifilum
Tychonema
Compactococcus
Aliterella
Gloeocapsopsis
Geitlerinema
Scytonemataceae
Nostoc
Chroococcales
Acaryochloridales
Nodosilineales
Pseudanabaenales
Tree scale: 0.1
