# Supplementary material for: Experiences of supporting primary and community healthcare workers affected by domestic abuse in the United Kingdom: A cross-sectional survey
Source: Eur J Gen Pract. 2025 Nov 10;31(1):2571600. doi: 10.1080/13814788.2025.2571600 (PMC12604119; doi:10.1080/13814788.2025.2571600)
Supplement: Supplemental Material [file tejp-2025-0043-File022.pdf › suppl_data/tejp-2025-0043-File022.pdf]

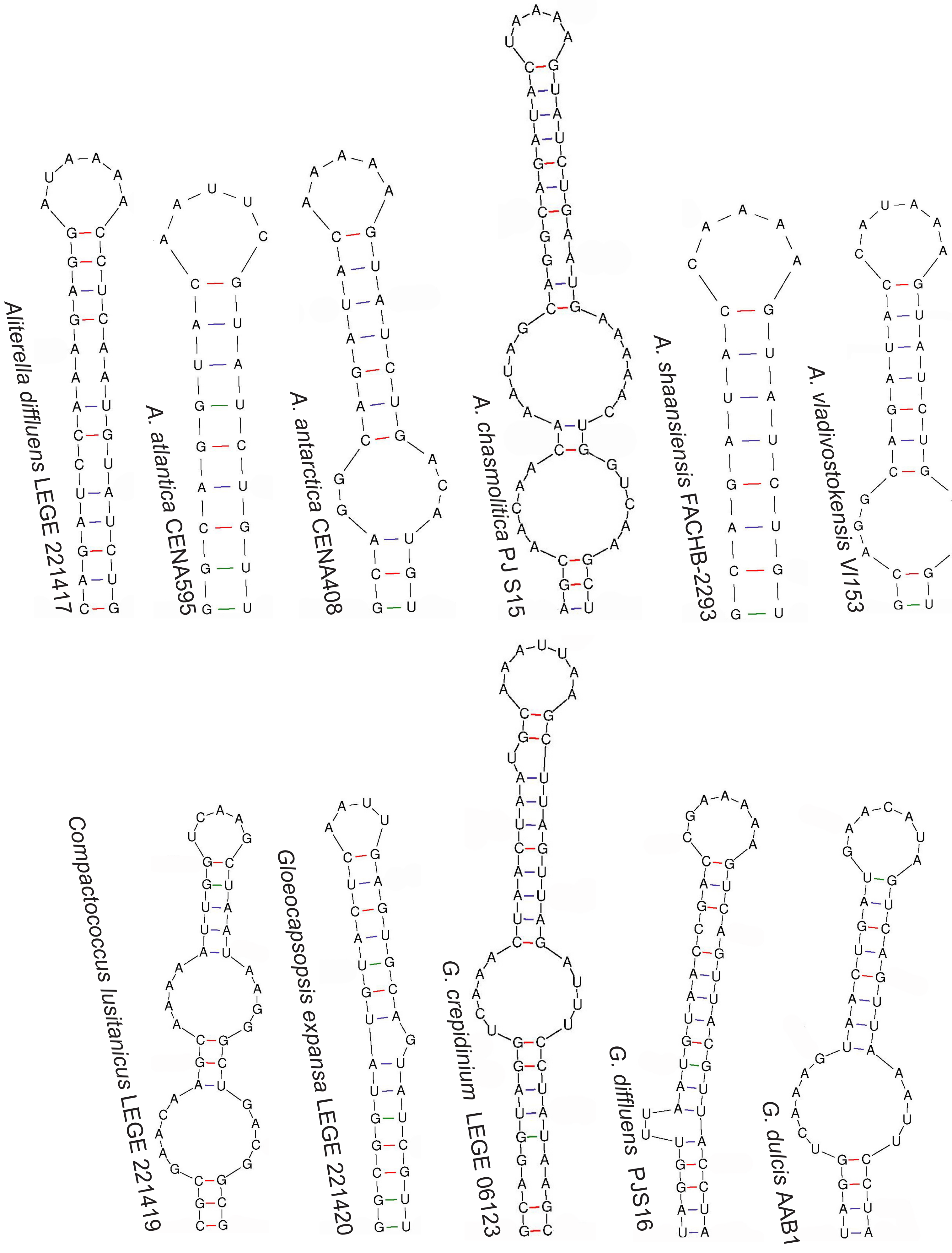

Aliterella diffluens LEGE 221417
A. atlantica CENA595
A. antarctica CENA408
A. chasmolitica PJ S15
A. shaansiensis FACHB-2293
A. vladivostokensis VI153
Compactococcus lusitanicus LEGE 221419
Gloeocapsopsis expansa LEGE 221420
G. crepidinium LEGE 06123
G. diffluens PJS16
G. dulcis AAB1
